# Medication Review and Enhanced Information Transfer at Discharge of Older Patients with Polypharmacy: a Cluster-Randomized Controlled Trial in Swiss Hospitals

**DOI:** 10.1007/s11606-022-07728-6

**Published:** 2022-08-31

**Authors:** Thomas Grischott, Yael Rachamin, Oliver Senn, Petra Hug, Thomas Rosemann, Stefan Neuner-Jehle

**Affiliations:** Institute of Primary Care, University of Zurich & University Hospital Zurich, Zurich, Switzerland

**Keywords:** communication, health care quality improvement, hospital medicine, medication safety, primary care

## Abstract

**Background:**

Medication safety in patients with polypharmacy at transitions of care is a focus of the current Third WHO Global Patient Safety Challenge. Medication review and communication between health care professionals are key targets to reduce medication-related harm.

**Objective:**

To study whether a hospital discharge intervention combining medication review with enhanced information transfer between hospital and primary care physicians can delay hospital readmission and impact health care utilization or other health-related outcomes of older inpatients with polypharmacy.

**Design:**

Cluster-randomized controlled trial in 21 Swiss hospitals between January 2019 and September 2020, with 6 months follow-up.

**Participants:**

Sixty-eight senior physicians and their blinded junior physicians included 609 patients ≥ 60 years taking ≥ 5 drugs.

**Interventions:**

Participating hospitals were randomized to either integrate a checklist-guided medication review and communication stimulus into their discharge processes, or follow usual discharge routines.

**Main Measures:**

Primary outcome was time-to-first-readmission to any hospital within 6 months, analyzed using a shared frailty model. Secondary outcomes covered readmission rates, emergency department visits, other medical consultations, mortality, drug numbers, proportions of patients with potentially inappropriate medication, and the patients’ quality of life.

**Key Results:**

At admission, 609 patients (mean age 77.5 (SD 8.6) years, 49.4% female) took a mean of 9.6 (4.2) drugs per patient. Time-to-first-readmission did not differ significantly between study arms (adjusted hazard ratio 1.14 (intervention vs. control arm), 95% CI [0.75–1.71], *p* = 0.54), nor did the 30-day hospital readmission rates (6.7% [3.3–10.1%] vs. 7.0% [3.6–10.3%]). Overall, there were no clinically relevant differences between study arms at 1, 3, and 6 months after discharge.

**Conclusions:**

The combination of a structured medication review with enhanced information transfer neither delayed hospital readmission nor improved other health-related outcomes of older inpatients with polypharmacy. Our results may help researchers in balancing practicality versus stringency of similar hospital discharge interventions.

**Study Registration:**

ISRCTN18427377, 10.1186/ISRCTN18427377

**Supplementary Information:**

The online version contains supplementary material available at 10.1007/s11606-022-07728-6.

## INTRODUCTION

Polypharmacy and inappropriate medication prescribing are major risk factors for adverse drug events, drug interactions, intake errors, and low medication adherence,^1–3^ thereby increasing morbidity, hospitalization rates, costs, and mortality in multimorbid older patients.^1,4,5^ Recognizing the rising prevalence of polypharmacy,^6^ national authorities and the WHO advocate deprescribing of inappropriate medications to reduce medication-related harm.^7–11^

Medication errors frequently occur at the interface between hospital-based and primary care,^12,13^ due to increased polypharmacy during hospitalization^14^ and communication deficits between hospital physicians (HPs) and primary care physicians (PCPs). In Switzerland, PCPs do not retain responsibility for the treatment of their hospitalized patients. Even when hospital treatment is delivered by independent specialists, and also in rehabilitation hospitals, general care is provided by HPs. When PCPs resume care after discharge, they are usually provided with a provisional discharge letter without reasons for medication changes, in which case they may tend to revert to the preadmission medication.^15^

Recent studies have shown that in-hospital medication reconciliation and review have the potential to reduce inappropriate prescribing and medication-related harm, but they alone seem insufficient to lastingly reduce polypharmacy and affect hard clinical outcomes.^16–21^ Although some complex, multi-component interventions that included discharge planning and outpatient follow-up resulted in lower readmission rates,^17,18^ others did not,^19–21^ and it remains unclear how to design an optimal discharge process aimed at minimizing readmission rates or emergency department (ED) visits.^22,23^

In this pragmatic study, we combined medication deprescribing at discharge with a communication stimulus between HPs and the patients’ PCPs. Our intent was to synthesize a minimal but sufficient subset of core components of existing discharge frameworks and best practice recommendations^24–26^ into a discharge procedure that could be flexibly adopted by different hospitals. Medication safety and communication were identified as a promising tandem, based on the rationale that better HP-PCP communication would foster consensus on appropriate discharge medication, and thereby lead to better anchored and longer-lasting maintenance of the optimized medication.^27^

The aim of this study was to evaluate whether such an approach reduces readmission rates of older inpatients with polypharmacy, compared to usual care, and whether it improves other health-related outcomes. Additional objectives addressed implementation issues, and related results have been published.^28^

## METHODS

The study was designed as an effectiveness-implementation hybrid trial set in hospitals in German-speaking Switzerland.^29^ Ethics approval was granted by the Ethics Committees Zurich and Bern, Switzerland (BASEC2018-00215). Here we present the effectiveness outcomes of this prospective, double-blind, cluster-randomized parallel-controlled trial following up hospitalized older patients with polypharmacy for 6 months after discharge.

### Recruitment, Allocation, and Patient Inclusion

In three waves (July 2018–October 2019), 165 medical decision-makers of all 141 non-psychiatric and non-pediatric hospitals in German-speaking Switzerland were contacted by postal mail. Ultimately, 21 hospitals chose to participate (with their internal medicine and/or geriatric wards) of which two were allocated as pilot hospitals to the intervention arm. Using blockwise covariate-constrained randomization^30^ (eMethods 1), the remaining 19 were 1:1 randomized to either intervention or control arm (February–November 2019).

**Figure 1:**
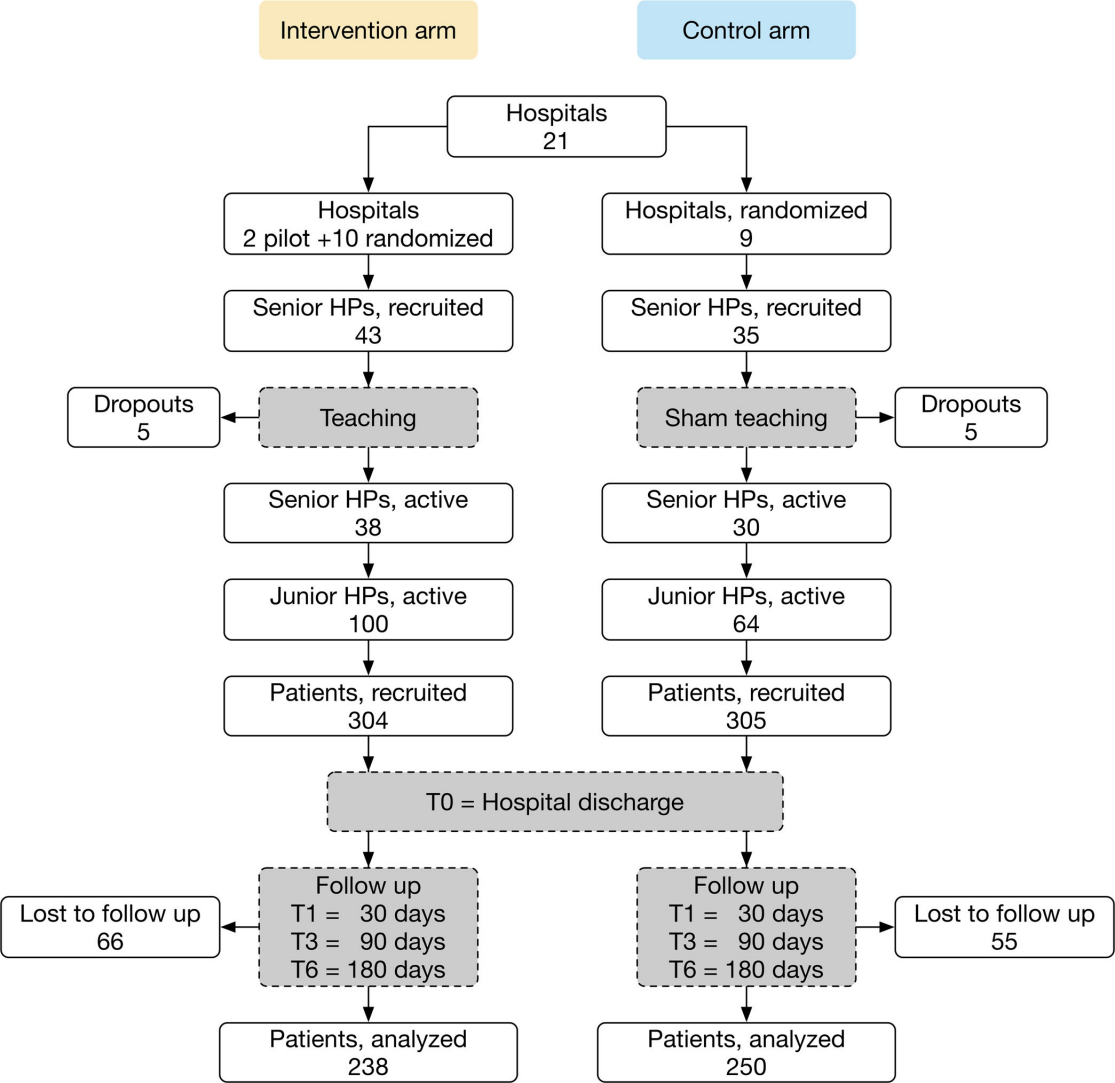
CONSORT diagram of the study design and participant flow. Abbreviation: HP, hospital physician.

January 2019–June 2020, 78 senior HPs (attending physicians), appointed by their superiors and consenting in writing to participate (without remuneration), were either instructed about the study intervention and data collection (intervention arm) or data collection alone (control arm). Several senior HPs withdrew from participation when the COVID-19 pandemic delayed the study in spring 2020. Finally, 38 senior HPs actively participated in the intervention and 30 in the control arm (Fig. 1). Their junior HPs (residents) were informed that their hospitals aimed to evaluate and improve discharge procedures, and thus remained blinded to treatment allocation. All participating junior HPs entered a raffle with a 1/10 chance of winning CHF 500 (USD 520).

January 2019–September 2020, both junior and senior HPs recruited patients. To minimize selection bias, HPs were asked to approach all eligible patients whenever feasible. Inclusion criteria were (1) in-hospital patient at the time of inclusion, (2) aged ≥ 60 years with ≥ 5 drugs prescribed (including “as needed” medication), and (3) written informed consent or consent from a legal representative. Patients were excluded in case of (1) end-stage disease with life expectancy < 3 months, or (2) cognitive inability to follow study procedures even with assistance. Patients were secondarily excluded when later transferred to another hospital or where medical care was otherwise not provided by a PCP. Readmitted study patients were acceptable as new index patients; however, this situation never occurred.

Soon after patient recruitment started, it became obvious that COVID-19 would drastically impact HP workload and recruitment success in virtually all participating hospitals. An ad hoc interim analysis revealed smaller-than expected clusters, a very low within-cluster correlation, and a relatively high fraction of censored data due to unexpectedly low baseline readmission rates, suggesting that our study would achieve 50–60% power with one-third of the target sample size. We also expected secondary outcomes to confirm a possible null result and thus mitigate this loss of power.

### Intervention

The intervention consisted of the instruction of a structured discharge procedure with two core components, namely (a) medication review performed by HPs and involving patients, and (b) measures to encourage medication-related information transfer between HPs and the referring PCPs:

- To optimize medication and deprescribe inappropriate drugs, each drug was subjected to questions about whether (i) the patient took it as prescribed, (ii) the drug’s indication was correct, (iii) the risk of side effects was lower than the expected benefit, (iv) the dose was correct given age and comorbidities, and (v) there was an alternative drug with a better benefit-to-risk ratio.
- To anchor medication changes with the follow-up PCPs, the discharge letters were customized. Depending on technical feasibility, they should contain (in order of preference) (i) a table of all medication changes with reasons (so implemented in *n* = 1 hospital), (ii) separate tables of admission and discharge medications, adjacent or in immediate sequence and again with reasons for changes (*n* = 8), or (iii) a table with the discharge medication only, and changes explained in the letter body (*n* = 3). Additionally, the discharge letters were supplemented by invitations to the PCPs to discuss the discharge medication with the responsible HPs (in bold print, with phone numbers of the discharging HPs).

In the 2-h instructions of the senior HPs by a member of the study team with extensive practical and research experience in polypharmacy and deprescribing in the primary care setting, they were first given background information on polypharmacy in multimorbid patients and then demonstrated the medication review using a hypothetical model patient. Subsequently, data collection was explained, and a discussion concluded the instructions.

Similar training of the junior HPs was entrusted to their seniors because of frequent personnel changes among the former. Both then participated in discharging study participants according to the study’s specifications. Proper implementation was guided by and recorded in a checklist by the discharging HPs (eFigure 1).

The senior HPs in the control arm were given a 2-h “sham” instruction by the same study team member, focusing on the significance of multimorbidity and polypharmacy, and on data collection. The untrained HPs in the control arm then discharged their patients as per the established local routines.

### Outcomes

The *primary* outcome was time-to-first-hospital-readmission within 6 months after discharge, and *secondary* outcomes covered readmission rates, numbers of ED visits, and other medical consultations (including with PCPs), all collected at 1, 3, and 6 months after discharge, as well as numbers and Anatomical Therapeutic Chemical (ATC) classes^31^ of drugs prescribed, proportions of patients with potentially inappropriate medications (PIMs), and the patients’ QoL, all collected at discharge and after 1, 3, and 6 months. Medications were classified as PIMs if they figured among the 2012 PRISCUS list^32^ or the updated 2019 Beers list of “unconditional PIMs”.^33^ QoL was assessed with the EQ-5D-3L instrument and evaluated using the French TTO value set.^34^ Additionally, baseline characteristics of hospitals, HPs, and patients were recorded (Table 1).

**Table 1:** Hospital, Participating HP, and Patient Characteristics

	Intervention arm	Control arm	SMD	Missing (%)^*^
Hospitals and HPs^†^				
Participating hospitals, no. (%)	12 (57.1)	9 (42.9)		
Acute care (vs. rehabilitation) hospital^‡^, no. (%)	9 (75.0)	7 (77.8)	0.07^‡^	0.0
Central (vs. local/basic care) hospital^§^, no. (%)	5 (41.7)	5 (55.6)	0.28^§^	0.0
Academic (vs. non-academic) hospitals, no. (%)	2 (16.7)	0 (0.0)	0.63	0.0
Hospitals with participating geriatric wards, no. (%)	2 (16.7)	0 (0.0)	0.63	0.0
No. of beds in participating wards^‖^, mean (SD)	64.75 (45.97)	69.44 (50.11)	0.10^‖^	0.0
No. of participating senior HPs, mean (SD)	3.17 (1.70)	3.33 (2.00)	0.09	0.0
No. of participating junior HPs, mean (SD)	8.33 (6.34)	7.11 (6.86)	0.18	0.0
No. of days of patient inclusion, mean (SD)	210.83 (89.47)	152.67 (58.99)	0.77	0.0
No. of patients included, mean (SD)	25.33 (16.57)	33.89 (23.84)	0.42	0.0
No. of patients lost to follow-up, mean (SD)	5.50 (5.76)	6.11 (3.33)	0.13	0.0
No. of patients analyzed, mean (SD)	19.83 (12.21)	27.78 (22.07)	0.45	0.0
Patients				
Patients included, no. (%)	304 (49.9)	305 (50.1)		
Male patients, no. (%)	151 (49.7)	157 (51.5)	0.04	0.0
Patients with legal representatives, no. (%)	6 (2.0)	2 (0.7)	0.12	0.0
Age, mean (SD)	77.77 (8.88)	77.12 (8.31)	0.08	0.0
No. of prescribed drugs at admission, mean (SD)	9.54 (4.00)	9.74 (4.37)	0.05	40.9

Abbreviations: *SMD*, standardized mean difference; *HPs*, hospital physicians; *SD*, standard deviation

^*^Fraction of missing data

^†^Means and SDs in the first section of the table are per hospital

^‡, §, ‖^Randomization covariates (^‡^hospital type, ^§^care level)

### Data Collection

Consent forms and copies of the discharge letters were sent to the study center in weekly batches, along with filled quality of life (QoL) questionnaires and, in the intervention arm, the checklists. During the study period and for 10 days beyond in each hospital, all contacts between PCPs and HPs were recorded by the discharging HP, other HPs, or administrative staff, with dates and duration. Six newsletters repeatedly reminded senior HPs to ensure that all PCP-HP contacts were recorded.

One (T1 = 30 days), 3 (T3 = 90 days), and 6 months (T6 = 180 days) after their discharge (T0), patients were requested by postal mail from the study center to report any hospital readmissions (for at least one night), ED visits, and other medical consultations (with reasons, if applicable) since their index discharge in paper-based case report forms (CRFs). The patients were also asked to provide their current medication plans and filled QoL forms. To improve data quality, a study nurse contacted patients or relatives in writing and/or by phone (up to three attempts) and also inquired with the patients’ PCPs for verifying patient reports and completing missing data.

Implementation outcomes were collected in parallel to the core study and evaluated according to an adapted framework^28^ for process evaluation studies.^35^

### Statistical Analysis

Hospital and patient characteristics and outcome baselines were presented as counts and proportions or means with standard deviations, with standardized mean differences and fractions of missing data.

Kaplan-Meier curves^36^ and the log rank test^37^ were used to compare the primary outcome (time-to-first-hospital-readmission) between study arms. The analysis was adjusted for hospital and patient characteristics using a shared frailty model^38^ with normally distributed cluster-specific random effects to account for within-hospital homogeneity.

Similar models were fitted for ED visits and other medical consultations. The competing risk death was treated as censor in the shared frailty models. Missing values (numbers of drugs at admission) were multiply imputed using R’s mice package,^39^ and for comparison, we also analyzed time-to-first-readmission on complete cases.

Additional secondary outcomes were presented with 95% confidence intervals (CIs) and/or compared between study arms using Fisher’s exact or Mann-Whitney *U* tests as appropriate. Sensitivity analyses as well as longitudinal analyses (numbers of drugs, QoL scores) using (generalized) linear mixed models are explained and presented with results in Online Supplement 1.

To quantify how multiple non-significant outcomes jointly speak against a true intervention effect, we calculated an approximate “aggregate power,” i.e., the probability of observing a hypothetic effect in at least one or two of *n* outcomes (eMethods 3).

All analyses of primary and secondary outcomes followed a modified intention-to-treat principle (Fig. 1) and were carried out using R, version 4.0.3.^40^

## RESULTS

In 21 participating hospitals, 68 senior and 164 junior HPs included 609 patients (intervention arm: 328 initial inclusions - 24 secondary exclusions; control arm: 317 - 12; *p* = 0.06). No patient dropped out before discharge, but 121 provided no follow-up data (intervention arm: 66; control arm: 55; *p* = 0.27) (Fig. 1). There were no significant differences in sex and age between those who did or did not complete follow-up.

On admission, the patients’ mean age was 77.5 years, they took a mean of 9.64 drugs, and 49.4% were female. Randomization covariates (hospital type, care level, number of beds in participating wards) and patient baseline characteristics were similar in both study arms (Table 1; eTable 1).

### Primary Outcome

**Figure 2:**
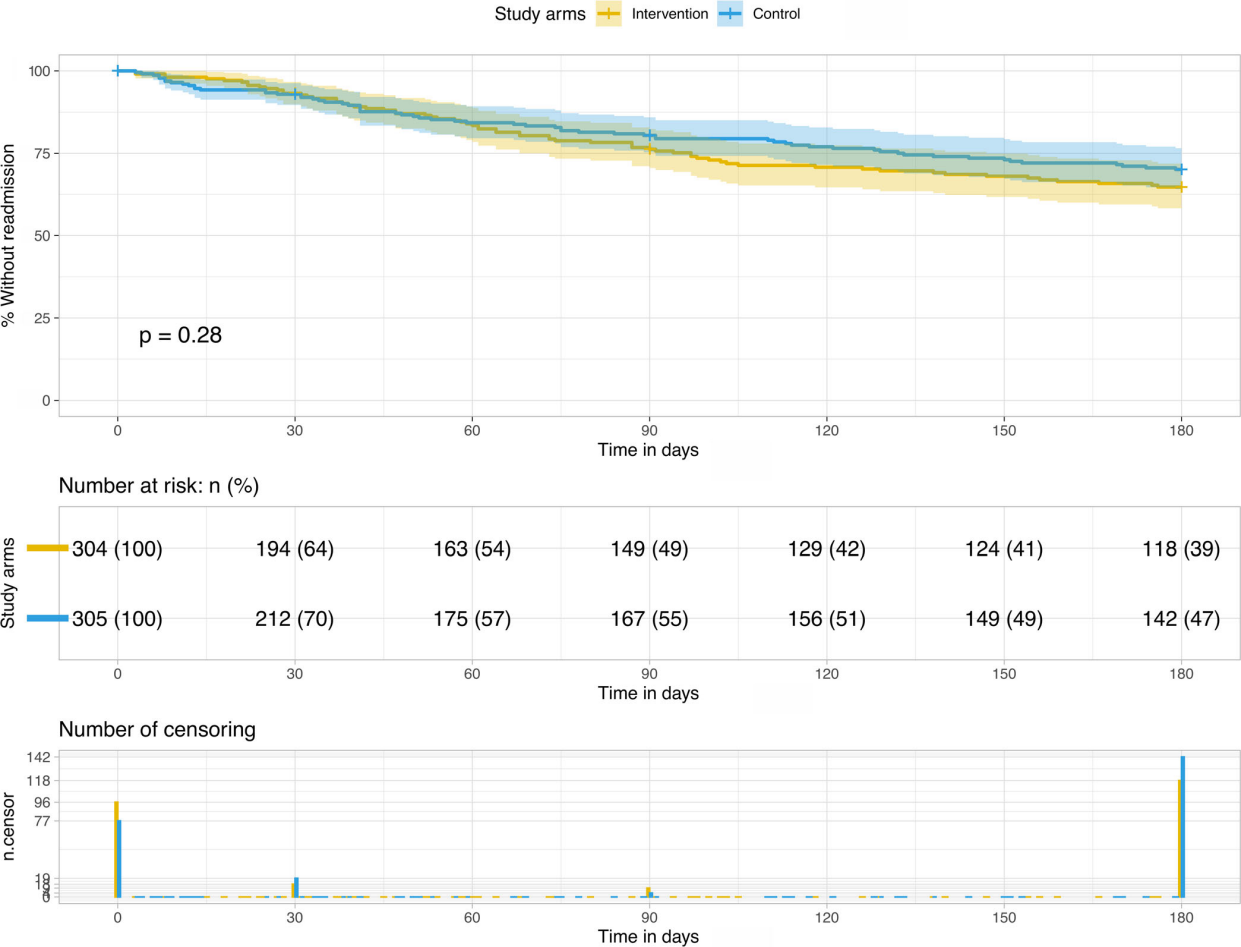
Time-to-first-readmission to any hospital within 180 days of the index discharge. Kaplan-Meier curve with log-rank test.

Time-to-first-readmission to any hospital within 6 months after discharge did not differ between study arms (Fig. 2; log rank test *p* = 0.28). The adjusted shared frailty model showed no significant discrepancy in hazard rates either (hazard ratio (HR) = 1.14 (intervention vs. control arm), 95% CI = [0.75-1.71], *p* = 0.54; eTable 2). Covariates reaching significance in the adjusted model were sex (HR = 1.50 (male vs. female), 95% CI = [1.05–2.13], *p* = 0.03), age (HR = 0.98 (per 5 year increase), 95% CI = [0.96–1.00], *p* = 0.03), and the number of drugs at admission (HR = 1.06 (per one additional drug), 95% CI = [1.01–1.12], *p* = 0.03).

### Secondary Outcomes

Table 2 presents rates of readmissions, ED visits (eFigure 2), and other medical consultations (eFigure 3) for each follow-up stage. All rates were within the CIs of the respective other study arm. Shared frailty models for time-to-ED visits and time-to-other medical consultations showed no significant differences between study arms (HR = 1.14, 95% CI = [0.63–2.09], *p* = 0.66; and HR = 1.11, 95% CI = [0.86–1.43], *p* = 0.42; eTables 4 and 5), and no consistent pattern regarding covariate influence.

**Table 2:** Rates (%) of Readmissions, ED Visits, and Other Medical Consultations, by Study Stage

Study stage	Readmissions		ED visits		Other medical consultations	
	Intervention arm	Control arm	Intervention arm	Control arm	Intervention arm	Control arm
T1 = 30 days	6.7 [3.3–10.1] (194)	7.0 [3.6–10.3] (212)	4.2 [1.1–7.1] (161)	6.9 [3.2–10.4] (176)	67.1 [58.6–73.9] (50)	70.0 [62.3–76.2] (53)
T3 = 90 days	23.7 [17.6–29.5] (149)	19.5 [14.1–24.7] (167)	12.3 [6.8–17.5] (119)	12.1 [7.1–16.9] (134)	94.8 [89.3–97.5] (8)	92.5 [87.1–95.6] (14)
T6 = 180 days	35.3 [28.1–41.7] (118)	29.9 [23.4–35.9] (142)	18.6 [14.2–28.3] (93)	18.6 [12.3–24.5] (113)	96.9 [91.4–98.9] (3)	95.5 [89.0–98.2] (3)

Abbreviations: *ED*, emergency department; […], 95% Wald confidence interval calculated on the log scale; (…), numbers of patients at risk

Any-cause mortality rates were 1.6% within 30 and 7.6% within 180 days after discharge and did not differ between study arms (eTable 6).

Table 3 summarizes the patients’ medications (eFigures 4 and 5), with total numbers and numbers of drugs per patient, proportions of patients with (at least one) PIM and of the drug classes oral antidiabetics, antihypertensives, statins, and acetylsalicylic acid, which were explicitly addressed as frequent candidates of inappropriate prescribing in the teaching sessions. Inspection of the CIs revealed neither differences between study arms nor temporal trends. In particular, a longitudinal mixed model confirmed the absence of a time trend in the number of drugs per patient (*p* = 0.07, 0.55, 0.63, and 0.29 for discharge and 1, 3, and 6 months later, each compared to admission; eTable 7).

**Table 3:**
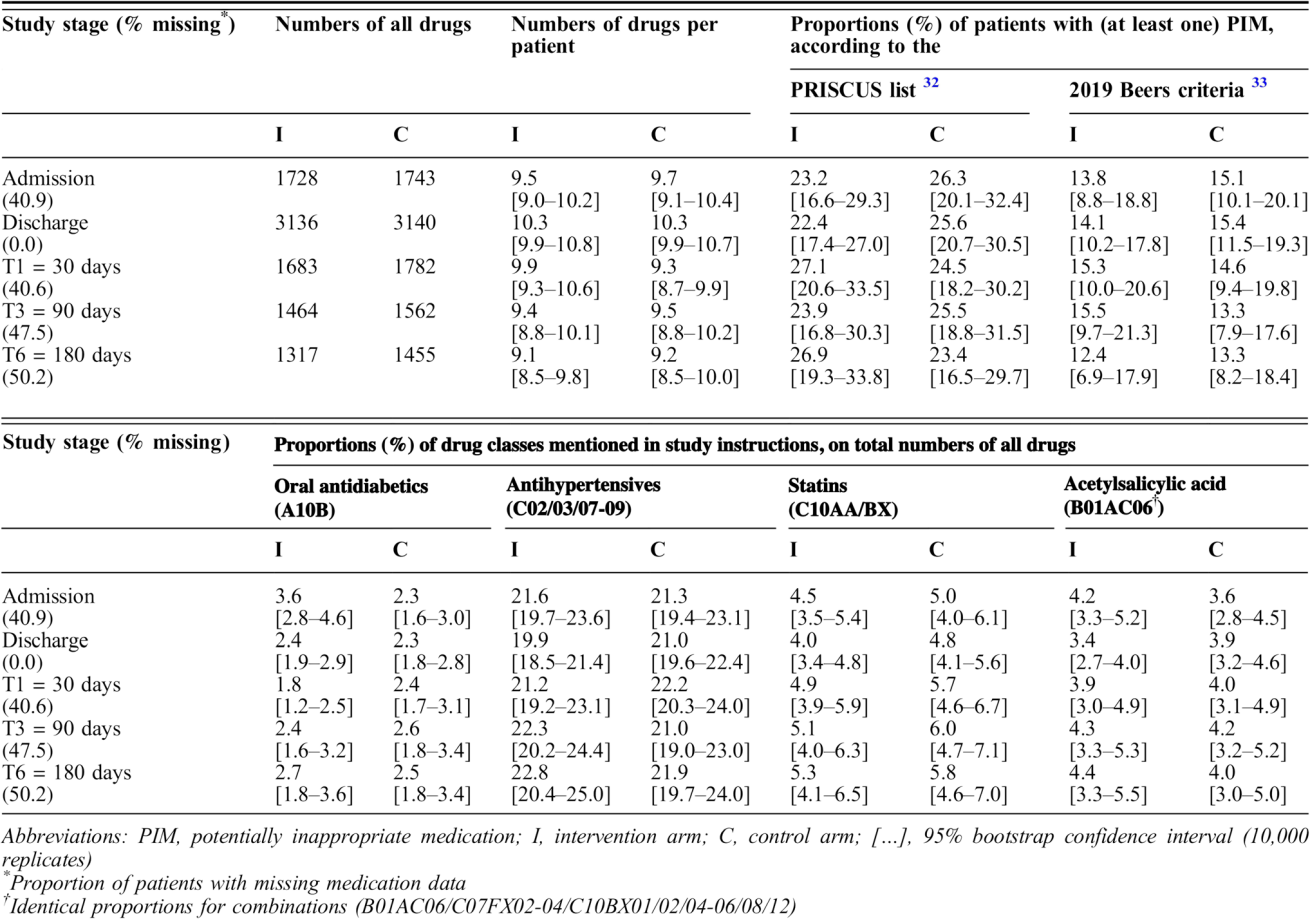
Medication, by Study Stage

eTables 8 and 9 in Online Supplement 1 analogously present proportions of all ATC anatomical main groups and of the three most frequently prescribed medications.

Regarding QoL, the raw data showed a slight increase over time, with some significant study arm differences (in favor of the control arm) at later time points (eTable 10). Imputation based on a plausible missing at random (MAR) assumption followed by fitting a longitudinal mixed model exposed raw *p* values < 0.05 as likely to be false positives due to attrition bias (eTable 11).

## DISCUSSION

In this pragmatic study, we combined medication review with a HP-PCP communication stimulus into a checklist-guided discharge procedure. This dual intervention did not decrease time-to-hospital-readmission of older inpatients with polypharmacy, compared with established discharge procedures, and showed no effect on secondary outcomes (apart from a slightly slower increase in QoL, likely due to attrition bias, and clinically irrelevant).

Previous research on the effectiveness of medication-related interventions at hospital discharge is inconclusive:^16,23^ Medication review alone was shown to reduce drug-related problems, but not readmission rates or mortality.^41–43^ In contrast, such effects were achieved in trials that involved patients beyond their discharge.^44–48^ Notably, a Swedish RCT with patients aged ≥ 80 years reported a 16% reduction of hospitalizations in the year following discharge,^17^ and in a Danish multicenter RCT, medication review plus individual follow-up by hospital pharmacists reduced readmissions within 6 months by 25%.^18^ Conversely, our study confirms the results of a recent crossover trial with 2644 patients from 4 Swedish hospitals that found no beneficial effects of medication review plus postdischarge patient follow-up on the incidence of unplanned hospital visits within 12 months, nor on health care utilization or mortality.^19^

Multiple reasons may account for this null result. Firstly, the baseline readmission rate in our sample (7.0% within 1 month; Table 2) was unexpectedly low, thus impeding further improvements. For example, US sources reported 30-day all-cause readmission rates of 13.9% in 2016,^49^ and in Swiss acute care hospitals, they were 11% across all ages in 2009–2016.^50^ A slight overrepresentation of smaller and rehabilitation hospitals in our sample may have resulted in a more favorable case mix with lower readmission risk.

Secondly, high-quality pre-existing discharge procedures and/or high postdischarge standards of care by ambulatory providers may have thwarted our intervention’s effect. Aside from the low baseline readmission rate, this hypothesis is supported by the observation that potential mediators of early readmission did not differ between study arms: (a) Early PCP support, inversely associated with readmission, ED visits, and mortality,^51,52^ was equally sought in both study arms (Table 2, other medical consultations). (b) Medication numbers increased just slightly (and insignificantly) from admission to discharge, when they were equal in both study arms (Table 3). (c) Likewise, there was no difference in the—relatively low^53^—fractions of patients with PIM, which is a well-known driver of hospitalization.^53^

Thirdly, encouraging PCPs to communicate with HPs—a key component of our intervention—may have failed. Few PCPs contacted HPs in the postdischarge period (4 documented contacts in the intervention and 10 in the control arm; *p* = 0.17). However, HP-initiated communication had much more frequently taken place during hospitalization (intervention arm: 22.4%, information missing for 36 patients; control arm 22.0%, missing for 30; *p* = 0.24), which further indicates a relatively high baseline quality of care.

The flexible implementation of our study minimally disrupted hospital routines,^28^ but possibly at the expense of a weaker intervention^54^ that generated no incremental benefit in hospitals with already well-organized discharge procedures including routine HP-PCP communication, critical medication review, and a high awareness of polypharmacy and deprescribing.

### Limitations

Our study suffered from under-recruitment in most hospitals during the first two waves of the COVID-19 pandemic in Switzerland, resulting in some loss of power. However, we assume a low risk of a false-negative result (Methods, eMethods 3).

The study design with a voluntary hospital sample and cluster-randomization before patient recruitment carried the risk of selection bias. HPs were instructed to invite all eligible patients to participate, but depending on workload, this was surely not always possible, and it is unclear how fluctuations in recruitment intensity may have affected the outcomes. Imperfect blinding (i.e., of junior HPs only), losses to follow-up, incomplete medication data, self-declared outcomes, and subjective measurement tools (e.g., for QoL) were other potential sources of bias.

Although our evaluation of the checklists suggested that the hospitals implemented the study largely as instructed, the COVID-19-related additional workload may at times have favored a somewhat leaner implementation on the HPs’ own initiative (e.g., cursory medication review).

Finally, we must assume that the HP-PCP communication in the postdischarge period, which we could not monitor reliably, was incompletely documented. That being said, follow-up surveys among PCPs confirmed that they often and readily adopted changes to their patients’ long-term medication without further consultation with HPs.^28^

## CONCLUSIONS

Our dual intervention combining a patient-centered medication review with enhanced medication-related information transfer between HPs and PCPs neither delayed hospital readmission nor impacted health care utilization, mortality, polypharmacy, or medication appropriateness among older inpatients after discharge. Given the consistency in all outcomes, we assume a true null result despite the limited sample size. Prioritizing flexibility over implementation rigor may have weakened the intervention. Our data and experience may help to strike an optimal balance between practicality and stringency in future hospital discharge interventions.

## Supplementary Information

ESM 1 (DOCX 1.45 mb)

ESM 2 (PDF 1.22 mb)

## Data Availability

The datasets generated and/or analyzed during the current study are available from the last author on reasonable request.
