# Supplementary material for: Medication Review and Enhanced Information Transfer at Discharge of Older Patients with Polypharmacy: a Cluster-Randomized Controlled Trial in Swiss Hospitals
Source: J Gen Intern Med. 2022 Aug 31;38(3):610–8. doi: 10.1007/s11606-022-07728-6 (PMC9432794; doi:10.1007/s11606-022-07728-6)
Supplement: Supplementary file 1 — (DOCX 1.45 mb) [file 11606_2022_7728_MOESM1_ESM.docx]

**ONLINE SUPPLEMENTARY MATERIAL**

Grischott T, Rachamin Y, Senn O, et al. Medication Review and Enhanced Information Transfer at Discharge of Older Patients with Polypharmacy—a Cluster-Randomized Controlled Trial in Swiss Hospitals. Journal of General Internal Medicine. 2022. doi: 10.1007/s11606-022-07728-6

**eMethods 1**. Randomization

**eMethods 2**. Multiple Imputation of Missing Data

**eMethods 3**. Power Considerations

**eTable 1**. Hospital and Participating HP Characteristics; Study and Inclusion Periods with Admissions per Hospital; Study population

**eTable 2**. Adjusted Imputed Shared Frailty Model for Time-to-Readmission

**eTable 3**. Adjusted Complete Case Shared Frailty Model for Time-to-Readmission

**eTable 4**. Adjusted Imputed Shared Frailty Model for Time-to-ED Visits

**eTable 5**. Adjusted Imputed Shared Frailty Model for Time-to-Other Medical Consultations

**eTable 6**. Mortality. Kaplan-Meier Estimates with 95% Wald CIs

**eTable 7**. Imputed Generalized Linear Mixed (“Poisson ANCOVA”) Model for Number of Drugs

**eTable 8**. Proportions of ATC Main Groups on the Total Numbers of Drugs Prescribed at Individual Study Stages

**eTable 9**. Proportions of Specific Drug Subclasses on the Total Numbers of Drugs Prescribed at Individual Study Stages, with 95% Bootstrap CIs

**eTable 10**. Quality of Life. Index Values and Visual Analogue Scale Scores of the EQ-5D-3L, with 95% Wald CIs

**eTable 11**. Imputed Linear Mixed (“ANCOVA”) Models of Quality of Life

**eFigure 1**. Discharge Checklist and Communication Stimulus

**eFigure 2**. Kaplan-Meier Plot of Emergency Department (ED) Visits

**eFigure 3**. Kaplan-Meier Plot of Other Medical Consultations

**eFigure 4**. ATC Therapeutic Subgroups at Discharge

**eFigure 5**. ATC Pharmacological Subgroups at Discharge

**eReferences**.

This online supplementary material has been provided by the authors to give readers additional information about their work. *eFigures 4 & 5 are intended for on-screen viewing in high magnification.*

**eMethods 1. Randomization**

The first two hospitals (H20, H21 in eTable 1) were designated as pilot hospitals. All other hospitals were randomized in three batches (first batch, allocated on 6 February 2019: H3, H4, H6-H9, H11, H13, H14, H17, H18; second batch, allocated on 2 April 2019: H1, H2, H10, H19; third batch, allocated on 26 November 2019: H5, H16, H12) by a member of the Institute of Primary Care using a covariate constrained randomization technique implemented in the “Shiny Balancer”.^1^ Covariates were hospital type (acute care vs. rehabilitation), care level (central vs. local/basic care) and the number of hospital beds in participating wards (as a proxy for hospital size and complexity). Covariate imbalance was measured by the metrics “1 - pValue of chi-squared test” (binary variables) and “1 - pValue of Kolmogorov-Smirnov test (integer variable)”, and equal weight was given to all three covariates. Each randomization round took the remaining imbalance from previous rounds into account. The later batches were formed smaller than planned in order not to delay the study start and patient recruitment in the hospitals already included.

**eMethods 2. Multiple Imputation of Missing Data**

Missing values (numbers of drugs at admission) were multiply imputed in *m* = 75 datasets under fully conditional specification using predictive mean matching with two-level imputation models (package mice 3.13.0^2^ with method 2l.pmm from package miceadds 3.11-6^3^ for all imputed variables) which included cluster means of metric variables as additional predictors and the Nelson-Aalen estimators of cumulative hazards instead of event times.

**eMethods 3. Power Considerations**

With 609 patients recruited, our study fell short of the target sample size of 2100, mainly due to the COVID-19 situation. This was partially mitigated by smaller-than-expected clusters and a very low within-cluster-correlation. Conversely, the loss of statistical power was accentuated by the relatively high proportion of censored data due to lower-than-expected baseline readmission rates. As we show below, these effects resulted in an overall relative loss of power of about 30%, which was again compensated for by the consistency of our results across all outcomes:

As detailed in the study protocol,^4^ we used Freedman's formula^5^ as given in Xie and Waksman^6^ to calculate the total number $n$ of subjects needed to detect a hazard reduction by 20% (i.e. a hazard ratio $HR = 0.8$) in the intervention with $\beta=80\%$ power:

$$n=\left( z_{\alpha/2}+z_{1-\beta} \right)^{2}\cdot\frac{\left( 1+HR \right)^{2}\cdot\left[ 1+\left( m-1 \right)\cdot\rho\right]}{\left( 1-HR \right)^{2}\cdot\left( 1-c \right)}$$

($z_{\alpha/2}$ and $z_{1-\beta}$ are quantiles of the standard normal distribution, $m$ ist the cluster size, $\rho$ the intra-cluster correlation, and $c$ the proportion censored.)

Assuming $m = 50$ and $\rho= 0.02$, we expected a design effect of $1 + (m-1)\cdot\rho= 1.98$. However, almost all clusters ended up smaller ($\bar{m}=29)$ than expected, and we also overestimated $\rho$. Overall, these effects were somewhat difficult to quantify since it is not straightforward to calculate $\rho$ from frailty models with log-normal frailty terms, but from an analogous model with a gamma distributed frailty term we estimated the variance of the random effect to be around $5\cdot{10}^{-7}$ which would then imply $\rho= {5\cdot{10}^{-7}}/\left( 5\cdot{10}^{-7}+2 \right)=2.5\cdot{10}^{-7}$(e.g. ^7^). Both effects, $\bar{m}=29$ and $\rho\approx0$, suggest that the true design effect was in fact close to 1, and half the planned sample size of 2100 participants would have sufficed to achieve $80\%$ power for the primary outcome.

Solving the above formula for $z_{1-\beta}$ yields

$z_{1-\beta}=\sqrt{\frac{n\cdot\left( 1-HR \right)^{2}\cdot\left( 1-c \right)}{\left( 1+HR \right)^{2}\cdot\left[ 1+\left( m-1 \right)\cdot\rho\right]}}-z_{\alpha/2}$.

With $n = 609$ patients, $m = \bar{m}=29$, $\rho=2.5\cdot{10}^{-7}$ and all other parameters as in the study protocol, we can therefore assume an effective power of $56.5\%$ not to overlook the hoped for (not the observed!) effect, corresponding to a relative loss of power of about 30%. However, these calculations are approximate, and they also disregard the fact that due to the lower-than-expected readmission rates, the fraction of censored event-times was higher and the number of events therefore lower than expected. The true loss of power will therefore be somewhat higher.

All previous considerations refer only to the primary outcome and ignore the fact that the non-significant secondary outcomes also speak for the ineffectiveness of our intervention. A simplified model calculation will illustrate how multiple outcomes contribute to power:

Assume that for suitably chosen alternative hypotheses of all statistical tests of $n-1$ independent secondary outcomes, all these tests have the same power of $1-\beta$. The “aggregated power”, i.e., the probability of “observing the intervention effect in at least one outcome”, is then $1-\beta^{n}$, and the probability of observing it in at least two outcomes is $1-\beta^{n}-n\cdot\beta^{n-1}\cdot(1-\beta)$. Counting ED visits, other medical consultations, mortality, numbers of drugs, proportions of patients with PIM, and the patients’ quality of life as secondary outcomes ($n=7$; there are more, actually, but these are the “most independent”; remember that we observed one single significant test result for quality of life) and assuming $1-\beta\approx50\%$, the two terms amount to roughly 99% and 94%, both well above the targeted power of 80% according to the study protocol.

**eTable 1. Hospital and Participating HP Characteristics; Study and Inclusion Periods with Admissions per Hospital; Study population**

| Hospital | Study arm | | Hospital characteristics | | | | | | Participating HPs | | | |  |
| --- | --- | --- | --- | --- | --- | --- | --- | --- | --- | --- | --- | --- | --- |
|  | | intervention | acute care | central | academic | internal | geriatric | n_beds | n_senior_HPs | n_male_sHPs | n_junior_HPs | n_male_jHPs | |
|  | |  |  |  |  |  |  |  |  |  |  |  | |
| H1 | | 1 | 0 | 0 | 0 | 1 | 0 | 70 | 3 | 1 | 4 | 3 | |
| H2 | | 0 | 1 | 1 | 0 | 1 | 0 | 130 | 0 | 0 | 6 | 4 | |
| H3 | | 0 | 1 | 1 | 0 | 1 | 0 | 75 | 2 | 2 | 4 | 1 | |
| H4 | | 1 | 1 | 0 | 0 | 1 | 0 | 40 | 3 | 2 | 6 | 2 | |
| H5 | | 1 | 0 | 0 | 0 | 1 | 1 | 180 | 2 | 1 | 0 | 0 | |
| H6 | | 0 | 1 | 0 | 0 | 1 | 0 | 15 | 3 | 2 | 4 | 1 | |
| H7 | | 1 | 1 | 0 | 0 | 1 | 0 | 28 | 3 | 1 | 8 | 2 | |
| H8 | | 0 | 0 | 0 | 0 | 1 | 0 | 65 | 5 | 3 | 1 | 1 | |
| H9 | | 0 | 0 | 0 | 0 | 1 | 0 | 167 | 7 | 5 | 5 | 2 | |
| H10 | | 0 | 1 | 1 | 0 | 1 | 0 | 30 | 4 | 1 | 11 | 6 | |
| H11 | | 0 | 1 | 1 | 0 | 1 | 0 | 60 | 3 | 3 | 24 | 11 | |
| H12 | | 1 | 1 | 1 | 1 | 1 | 0 | 55 | 2 | 2 | 4 | 2 | |
| H13 | | 1 | 1 | 0 | 0 | 1 | 0 | 50 | 7 | 6 | 14 | 4 | |
| H14 | | 1 | 1 | 1 | 0 | 1 | 1 | 125 | 3 | 2 | 20 | 6 | |
| H15 | | 0 | 1 | 0 | 0 | 1 | 0 | 23 | 4 | 2 | 4 | 3 | |
| H16 | | 0 | 1 | 1 | 0 | 1 | 0 | 60 | 2 | 1 | 5 | 2 | |
| H17 | | 1 | 1 | 0 | 0 | 1 | 0 | 23 | 2 | 1 | 7 | 4 | |
| H18 | | 1 | 0 | 0 | 0 | 1 | 0 | 36 | 1 | 1 | 5 | 1 | |
| H19 | | 1 | 1 | 1 | 0 | 1 | 0 | 80 | 3 | 1 | 7 | 3 | |
| H20 | | 1 | 1 | 1 | 0 | 1 | 0 | 60 | 6 | 4 | 20 | 14 | |
| H21 | | 1 | 1 | 1 | 1 | 1 | 0 | 30 | 3 | 2 | 5 | 3 | |
| I | | 12 | 9 | 5 | 2 | 12 | 2 | 777 | 38 | 24 | 100 | 44 | |
| C | | 9 | 7 | 5 | 0 | 9 | 0 | 625 | 30 | 19 | 64 | 31 | |
| T | | 21 | 16 | 10 | 2 | 21 | 2 | 1402 | 68 | 43 | 164 | 75 | |
|  | |  |  |  |  |  |  |  |  |  |  |  | |
| Hospital | | Study arm | Study period | | | Inclusion period | | | | Patients included | | |  |
|  | | intervention | sHP_instruction | hosp_dismissal | n_days_study | first_out | last_out | n_days_inclusion | n_admitted | n_included | n_lost_to_fup | n_patients | |
|  | |  |  |  |  |  |  |  |  |  |  |  | |
| H1 | | 1 | 05.24.19 | 03.23.20 | 304 | 06.24.19 | 11.17.19 | 146 | 160 | 12 | 2 | 10 | |
| H2 | | 0 | 07.11.19 | 03.23.20 | 256 | 08.09.19 | 11.14.19 | 97 | 729 | 21 | 6 | 15 | |
| H3 | | 0 | 05.17.19 | 03.23.20 | 311 | 08.17.19 | 12.23.19 | 128 | 850 | 21 | 3 | 18 | |
| H4 | | 1 | 03.20.19 | 03.23.20 | 369 | 04.26.19 | 03.16.20 | 325 | 263 | 8 | 2 | 6 | |
| H5 | | 1 | 12.10.19 | 09.30.20 | 295 | 02.18.20 | 09.01.20 | 196 | 1188 | 20 | 3 | 17 | |
| H6 | | 0 | 05.24.19 | 03.23.20 | 304 | 05.28.19 | 02.07.20 | 255 | 349 | 31 | 11 | 20 | |
| H7 | | 1 | 02.28.19 | 02.14.20 | 351 | 06.21.19 | 02.15.20 | 239 | 579 | 18 | 2 | 16 | |
| H8 | | 0 | 05.09.19 | 03.23.20 | 319 | 07.27.19 | 02.10.20 | 198 | 200 | 15 | 2 | 13 | |
| H9 | | 0 | 03.26.19 | 02.14.20 | 325 | 04.13.19 | 11.14.19 | 215 | 477 | 49 | 4 | 45 | |
| H10 | | 0 | 11.27.19 | 03.23.20 | 117 | 12.21.19 | 03.24.20 | 94 | 455 | 37 | 9 | 28 | |
| H11 | | 0 | 05.14.19 | 02.14.20 | 276 | 07.09.19 | 12.24.19 | 168 | 444 | 89 | 10 | 79 | |
| H12 | | 1 | 03.09.20 | 09.30.20 | 205 | 03.13.20 | 09.29.20 | 200 | 1106 | 21 | 4 | 17 | |
| H13 | | 1 | 05.10.19 | 03.11.20 | 306 | 06.06.19 | 01.10.20 | 218 | 773 | 37 | 4 | 33 | |
| H14 | | 1 | 05.21.19 | 03.23.20 | 307 | 06.20.19 | 03.17.20 | 271 | 1952 | 54 | 9 | 45 | |
| H15 | | 0 | 06.11.19 | 03.23.20 | 286 | 07.12.19 | 11.11.19 | 122 | 301 | 10 | 3 | 7 | |
| H16 | | 0 | 06.04.20 | 09.30.20 | 118 | 06.18.20 | 09.23.20 | 97 | NA | 32 | 7 | 25 | |
| H17 | | 1 | 03.15.19 | 03.05.20 | 356 | 03.30.19 | 01.28.20 | 304 | NA | 50 | 19 | 31 | |
| H18 | | 1 | 02.15.19 | 02.14.20 | 364 | 04.06.19 | 07.12.19 | 97 | 170 | 11 | 0 | 11 | |
| H19 | | 1 | 06.21.19 | 03.23.20 | 276 | 07.24.19 | 08.16.19 | 23 | 174 | 8 | 0 | 8 | |
| H20 | | 1 | 01.25.19 | 02.14.20 | 385 | 02.11.19 | 12.14.19 | 306 | 1493 | 44 | 13 | 31 | |
| H21 | | 1 | 03.20.19 | 02.14.20 | 331 | 04.11.19 | 11.02.19 | 205 | 297 | 21 | 8 | 13 | |
| I | | 12 |  |  | 3849 |  |  | 2530 | 8155 | 304 | 66 | 238 | |
| C | | 9 |  |  | 2312 |  |  | 1374 | 3805 | 305 | 55 | 250 | |
| T | | 21 |  |  | 6161 |  |  | 3904 | 11960 | 609 | 121 | 488 | |

Abbreviations: HP, hospital physician;

n_beds, number of beds in participating ward(s);

n_senior_HPs, number of actively participating male senior HPs;

n_male_sHPs, number of actively participating senior HPs;

n_junior_HPs, number of participating male junior HPs;

n_male_jHPs, number of participating junior HPs;

sHP_instruction, date of senior HP instruction (mm.dd.yy);

hosp_dismissal, date of recruitment termination (mm.dd.yy);

n_days_study, number of days from sHP_instruction until hosp_dismissal;

first_out, discharge date of first study patient (mm.dd.yy);

last_out, discharge date of last study patient (mm.dd.yy);

n_days_inclusion, number of days from first_out until last_out;

n_admitted, number of inpatients ≥60 years in (or projected for) inclusion period (including patients with <5 drugs or otherwise not eligible);

n_included, number of initially included – number of secondarily excluded patients;

n_lost_to_fup, number of patients lost to follow-up (no follow-up data available);

n_patients, number of patients analyzed;

I, intervention arm; C, control arm; T, total.

**eTable 2. Adjusted Imputed Shared Frailty Model for Time-to-Readmission**

Model (using R’s coxme notation):

coxme(Surv(Time-to-readmission, Readmission_event) ~ Study_arm (reference level = control arm) + Patient_sex (reference level = female) + Patient_age (reference level = 60) + Legal_representation (reference level = no) + Number_of_drugs_at_admission (reference level = 5) + Hospital_type (reference level = rehabilitation hospital) + Care_level (reference level = local/basic care) + Academic_hospital (reference level = no) + Number_of_beds (centered around mean) + (1 | Hospital_identifier))

| Fixed coefficients: | Estimate | 95% Wald CI | *p* |
| --- | --- | --- | --- |
| Study arm (intervention) | **1.14** | [0.75-1.71] | 0.54 |
| Patient sex (male) | 1.50 | [1.05-2.13] | 0.03 |
| Patient age (per year over 60) | 0.98 | [0.96-1.00] | 0.03 |
| Legal representation (yes) | 2.38 | [0.55-10.25] | 0.24 |
| Number of drugs at admission (per drug > 5) | 1.06 | [1.01-1.12] | 0.03 |
| Hospital type (acute care) | 2.17 | [0.90-5.23] | 0.09 |
| Care level (central) | 0.98 | [0.57-1.69] | 0.93 |
| Academic hospital (yes) | 1.07 | [0.49-2.32] | 0.86 |
| Number of beds (per bed) | 1.00 | [1.00-1.01] | 0.49 |

**eTable 3. Adjusted Complete Case Shared Frailty Model for Time-to-Readmission**

Model: As above.

| Fixed coefficients: | Estimate | 95% Wald CI | *p* |
| --- | --- | --- | --- |
| Study arm (intervention) | **0.94** | [0.54-1.61] | 0.81 |
| Patient sex (male) | 1.77 | [1.12-2.79] | 0.01 |
| Patient age (per year over 60) | 0.97 | [0.95-1.00] | 0.03 |
| Legal representation (yes) | 2.97 | [0.40-22.01] | 0.29 |
| Number of drugs at admission (per drug > 5) | 1.07 | [1.02-1.13] | 0.00 |
| Hospital type (acute care) | 1.65 | [0.65-4.23] | 0.29 |
| Care level (central) | 0.82 | [0.42-1.59] | 0.56 |
| Academic hospital (yes) | 0.97 | [0.35-2.68] | 0.96 |
| Number of beds (per bed) | 1.01 | [1.00-1.01] | 0.16 |

Note that entry medication was missing for 40.9% of patients (Table 3). A sensitivity analysis showed that imputation had a similar effect on the HR estimate as replacing the admission medication (in the model underlying eTable 2) by the complete and highly correlated discharge medication (*ρ* = 0.78 on complete cases): Adjusted HR = **1.12**, 95% Wald CI = [0.75-1.67], *p* = 0.59.

A Cox proportional hazards model (analogous to the model underlying eTable 2, imputed but without random effect) yielded similar results, too, indicating a low clustering effect: Adjusted HR = **1.13**, 95% Wald CI = [0.76-1.70], p = 0.55.

Models for time-to-emergency and time-to-other medical consultations:

**eTable 4. Adjusted Imputed Shared Frailty Model for Time-to-ED Visits**

Model (using R’s coxme notation):

coxme(Surv(Time-to-emergency, Emergency_event) ~ Study_arm (reference level = control arm) + Patient_sex (reference level = female) + Patient_age (reference level = 60) + Legal_representation (reference level = no) + Number_of_drugs_at_admission (reference level = 5) + Hospital_type (reference level = rehabilitation hospital) + Care_level (reference level = local/basic care) + Academic_hospital (reference level = no) + Number_of_beds (centered around mean) + (1 | Hospital_identifier))

| Fixed coefficients: | Estimate | 95% Wald CI | *p* |
| --- | --- | --- | --- |
| Study arm (intervention) | **1.14** | [0.63-2.09] | 0.66 |
| Patient sex (male) | 1.09 | [0.65-1.84] | 0.74 |
| Patient age (per year over 60) | 0.97 | [0.94-1.01] | 0.10 |
| Legal representation (yes) | 2.59 | [0.34-19.50] | 0.36 |
| Number of drugs at admission (per drug > 5) | 1.02 | [0.95-1.09] | 0.64 |
| Hospital type (acute care) | 1.86 | [0.56-6.19] | 0.31 |
| Care level (central) | 1.15 | [0.50-2.64] | 0.74 |
| Academic hospital (yes) | 0.69 | [0.21-2.24] | 0.53 |
| Number of beds (per bed) | 1.00 | [0.99-1.01] | 0.85 |

**eTable 5. Adjusted Imputed Shared Frailty Model for Time-to-Other Medical Consultations**

Model (using R’s coxme notation):

coxme(Surv(Time-to-consultation, Consultation_event) ~ Study_arm (reference level = control arm) + Patient_sex (reference level = female) + Patient_age (reference level = 60) + Legal_representation (reference level = no) + Number_of_drugs_at_admission (reference level = 5) + Hospital_type (reference level = rehabilitation hospital) + Care_level (reference level = local/basic care) + Academic_hospital (reference level = no) + Number_of_beds (centered around mean) + (1 | Hospital_identifier))

| Fixed coefficients: | Estimate | 95% Wald CI | *p* |
| --- | --- | --- | --- |
| Study arm (intervention) | **1.11** | [0.86-1.43] | 0.42 |
| Patient sex (male) | 0.96 | [0.76-1.21] | 0.74 |
| Patient age (per year over 60) | 0.99 | [0.98-1.01] | 0.49 |
| Legal representation (yes) | 4.62 | [1.43-14.93] | 0.01 |
| Number of drugs at admission (per drug > 5) | 0.99 | [0.96-1.02] | 0.51 |
| Hospital type (acute care) | 1.27 | [0.78-2.06] | 0.34 |
| Care level (central) | 0.93 | [0.65-1.33] | 0.69 |
| Academic hospital (yes) | 0.54 | [0.32-0.93] | 0.03 |
| Number of beds (per bed) | 1.00 | [1.00-1.00] | 0.64 |

**eTable 6. Mortality. Kaplan-Meier Estimates (%) with 95% Wald CIs** (calculated on the log scale)

| Intervention arm | Control arm |  |
| --- | --- | --- |
| 0.0 [0.0-0.0] | 0.4 [0.0-1.2] | within 7 days after discharge, |
| 1.3 [0.0-2.7] | 1.6 [0.0-3.1] | within 30 days, |
| 5.5 [2.6-8.4] | 4.5 [1.9-7.0] | within 90 days, and |
| 7.8 [4.3-11.2] | 7.5 [4.1-10.7] | within 180 days. |

**eTable 7. Imputed Generalized Linear Mixed (“Poisson ANCOVA”) Model for Number of Drugs**

Model (using R’s glmer notation):

glmer(Number of drugs (reference level = 5) ~ Study_stage (reference level = admission) + Study_arm (reference level = control arm):Study_stage + (1 | Hospital_identifier / Patient_identifier), family = poisson)

| Coefficients: | Estimate | 95% Wald CI | *p* |
| --- | --- | --- | --- |
| (Intercept) | 9.33 | [8.77-9.92] | 0.00 |
| Discharge | 1.06 | [0.99-1.13] | 0.07 |
| T1 | 0.98 | [0.91-1.05] | 0.55 |
| T3 | 0.98 | [0.92-1.05] | 0.63 |
| T6 | 0.96 | [0.89-1.03] | 0.29 |
| Discharge:Study arm (intervention) | 1.01 | [0.93-1.10] | 0.76 |
| T1:Study arm (intervention) | 1.05 | [0.96-1.16] | 0.29 |
| T3:Study arm (intervention) | 1.01 | [0.91-1.12] | 0.88 |
| T6:Study arm (intervention) | 1.01 | [0.90-1.13] | 0.90 |

**eTable 8. Proportions (%) of ATC Main Groups on the Total Numbers of Drugs Prescribed at Individual Study Stages**

| Study stage | Number of all drugs | | A | | B | | C | | D | |
| --- | --- | --- | --- | --- | --- | --- | --- | --- | --- | --- |
|  | I | C | I | C | I | C | I | C | I | C |
| Admission | 1728 | 1743 | 23.4 | 24.0 | 11.8 | 10.7 | 29.0 | 28.5 | 1.3 | 1.1 |
| Discharge | 3136 | 3140 | 24.0 | 25.0 | 11.0 | 12.0 | 26.4 | 28.7 | 1.1 | 0.8 |
| T1 = 30 days | 1683 | 1782 | 22.7 | 23.8 | 11.7 | 12.6 | 28.9 | 30.9 | 1.2 | 1.0 |
| T3 = 60 days | 1464 | 1562 | 22.5 | 23.3 | 11.4 | 12.2 | 30.2 | 29.7 | 0.9 | 1.3 |
| T6 = 180 days | 1317 | 1455 | 21.0 | 23.2 | 11.5 | 12.2 | 31.4 | 30.9 | 0.5 | 1.7 |
|  | G | | H | | J | | L | | M | |
|  | I | C | I | C | I | C | I | C | I | C |
| Admission | 3.0 | 2.5 | 2.8 | 2.6 | 1.5 | 1.4 | 1.4 | 1.2 | 3.1 | 4.5 |
| Discharge | 2.7 | 2.4 | 3.1 | 3.1 | 2.6 | 1.6 | 1.1 | 0.9 | 3.4 | 3.9 |
| T1 = 30 days | 2.9 | 2.4 | 3.0 | 2.8 | 1.5 | 1.0 | 1.3 | 1.0 | 3.7 | 4.6 |
| T3 = 60 days | 2.7 | 2.4 | 3.7 | 2.8 | 1.2 | 1.5 | 1.2 | 1.3 | 3.8 | 5.3 |
| T6 = 180 days | 2.9 | 2.9 | 3.5 | 2.7 | 1.7 | 1.1 | 1.0 | 1.2 | 4.2 | 5.9 |
|  | N | | P | | R | | S | | V | |
|  | I | C | I | C | I | C | I | C | I | C |
| Admission | 12.6 | 14.0 | 0.1 | 0.0 | 8.0 | 7.1 | 1.5 | 1.7 | 0.8 | 0.7 |
| Discharge | 15.3 | 14.0 | 0.0 | 0.1 | 7.0 | 5.7 | 1.5 | 1.4 | 0.8 | 0.6 |
| T1 = 30 days | 13.5 | 12.9 | 0.0 | 0.1 | 7.3 | 5.3 | 1.7 | 1.4 | 0.6 | 0.4 |
| T3 = 60 days | 12.6 | 11.9 | 0.0 | 0.2 | 7.9 | 5.9 | 1.6 | 1.5 | 0.5 | 0.6 |
| T6 = 180 days | 11.5 | 11.3 | 0.0 | 0.1 | 8.9 | 5.2 | 1.6 | 1.4 | 0.4 | 0.2 |

Abbreviations: I, intervention arm; C, control arm; A = alimentary tract and metabolism; B, blood and blood forming organs; C, cardiovascular system; D, dermatologicals; G, genito-urinary system and sex hormones; H, systemic hormonal preparations, excl. sex hormones and insulins; J, antiinfectives for systemic use; L, antineoplastic and immunomodulating agents; M, musculo-skeletal system; N, nervous system; P, antiparasitic products, insecticides and repellents; R, respiratory system; S, sensory organs; V, various.

**eTable 9. Proportions (%) of Most Frequent Drugs on the Total Numbers of Drugs Prescribed at Individual Study Stages, with 95% Bootstrap CIs**

| Study stage  (% missing^*^) | Number of all drugs | | Torasemide | | Pantoprazole | | Acetylsalicylic acid | |
| --- | --- | --- | --- | --- | --- | --- | --- | --- |
|  | I | C | I | C | I | C | I | C |
| Admission  (40.9) | 1728 | 1743 | 5.0  [3.9-6.0] | 4.3  [3.3-5.2] | 4.2  [3.2-5.2] | 4.5  [3.6-5.5] | 4.2  [3.3-5.2] | 3.6  [2.8-4.5] |
| Discharge  (0.0) | 3136 | 3140 | 4.6  [3.8-5.3] | 4.1  [3.4-4.8] | 4.2  [3.5-4.9] | 4.8  [4.1-5.6] | 3.4  [2.7-4.0] | 3.9  [3.2-4.6] |
| T1 = 30 days  (40.6) | 1683 | 1782 | 4.7  [3.7-5.8] | 3.9  [3.0-4.8] | 4.2  [3.3-5.2] | 4.8  [3.8-5.8] | 3.9  [3.0-4.9] | 4.0  [3.1-4.9] |
| T3 = 60 days  (47.5) | 1464 | 1562 | 4.8  [3.7-5.9] | 4.0  [3.1-5.0] | 4.4  [3.3-5.5] | 4.9  [3.8-6.0] | 4.3  [3.3-5.3] | 4.2  [3.2-5.2] |
| T6 = 180 days  (50.2) | 1317 | 1455 | 4.7  [3.6-5.9] | 3.8  [2.9-4.9] | 4.8  [3.6-5.9] | 4.6  [3.6-5.7] | 4.4  [3.3-5.5] | 4.0  [3.0-5.0] |

Abbreviations: I, intervention arm; C, control arm; […], 95% bootstrap confidence interval (10’000 replicates).

^*^ Proportion of patients with missing medication data.

**eTable 10. Quality of Life. Index Values (calculated using the French TTO value set) and Visual Analogue Scale (VAS) Scores of the EQ-5D-3L, with 95% Bootstrap CIs and *U* test *p* values**

| Study stage | TTO | | | | VAS | | | |
| --- | --- | --- | --- | --- | --- | --- | --- | --- |
|  | I | C | *p* | Missing (%)* | I | C | *p* | Missing (%)* |
| Discharge | 0.68 [0.64-0.71] | 0.67 [0.63-0.70] | 0.51 | 7.2 | 63.4 [61.3-65.6] | 66.9 [64.8-68.9] | 0.03 | 6.4 |
| T1 = 30 days | 0.65 [0.60-0.69] | 0.72 [0.69-0.76] | 0.01 | 44.7 | 64.8 [61.8-67.7] | 69.7 [67.2-72.2] | 0.02 | 41.4 |
| T3 = 90 days | 0.68 [0.63-0.72] | 0.74 [0.69-0.78] | 0.03 | 51.1 | 67.5 [64.6-70.3] | 71.7 [68.8-74.4] | 0.03 | 48.4 |
| T6 = 180 days | 0.68 [0.64-0.72] | 0.74 [0.70-0.79] | 0.01 | 51.7 | 67.4 [64.2-70.6] | 75.4 [72.7-78.0] | <0.001 | 50.6 |

Abbreviations: TTO, time trade-off; VAS, EQ-5D-visual analogue scale; I, intervention arm; C, control arm; […], 95% bootstrap confidence interval (10’000 replicates).

^*^ Proportion of patients with missing QoL data.

Analysis of repeated QoL measures by means of a linear mixed (“ANCOVA”) model on multiply imputed data revealed a weak time trend but exposed most of the low *p* values in the above eTable 12 (study stages T1 and T6) as probable false positives originating from MAR (missing at random) QoL data. The significant interaction at T1 in the TTO model (in eTable 13 below) could be a random result due to multiple testing.

**eTable 11. Imputed Linear Mixed (“ANCOVA”) Models of Quality of Life**

TTO Model (using R’s lmer notation):

lmer(Quality_of_life_TTO ~ Study_stage (reference level = discharge) + Study_arm (reference level = control arm):Study_stage + (1 | Hospital_identifier / Patient_identifier))

| Fixed effects: | Estimate | 95% Wald CI | *p* |
| --- | --- | --- | --- |
| (Intercept) | 0.667 | [0.634-0.700] | 0.00 |
| T1 | 0.042 | [0.001-0.082] | 0.04 |
| T3 | 0.047 | [0.004-0.090] | 0.03 |
| T6 | 0.056 | [0.013-0.098] | 0.01 |
| T1:Study arm (intervention) | -0.066 | [-0.125-(-0.006)] | 0.03 |
| T3:Study arm (intervention) | -0.049 | [-0.110-0.013] | 0.12 |
| T6:Study arm (intervention) | -0.049 | [-0.109-0.010] | 0.10 |

VAS Model (using R’s lmer notation):

lmer(Quality_of_life_VAS ~ Study_stage (reference level = discharge) + Study_arm (reference level = control arm):Study_stage + (1 | Hospital_identifier / Patient_identifier))

| Fixed effects: | Estimate | 95% Wald CI | *p* |
| --- | --- | --- | --- |
| (Intercept) | 64.8 | [62.5-67.2] | 0.00 |
| T1 | 2.9 | [0.3-5.5] | 0.03 |
| T3 | 4.6 | [1.8-7.4] | 0.00 |
| T6 | 7.1 | [4.2-9.9] | 0.00 |
| T1:Study arm (intervention) | -2.3 | [-6.1-1.5] | 0.23 |
| T3:Study arm (intervention) | -1.9 | [-6.0-2.2] | 0.37 |
| T6:Study arm (intervention) | -3.2 | [-7.2-0.7] | 0.11 |

**eFigure 1. Discharge Checklist and Communication Stimulus**


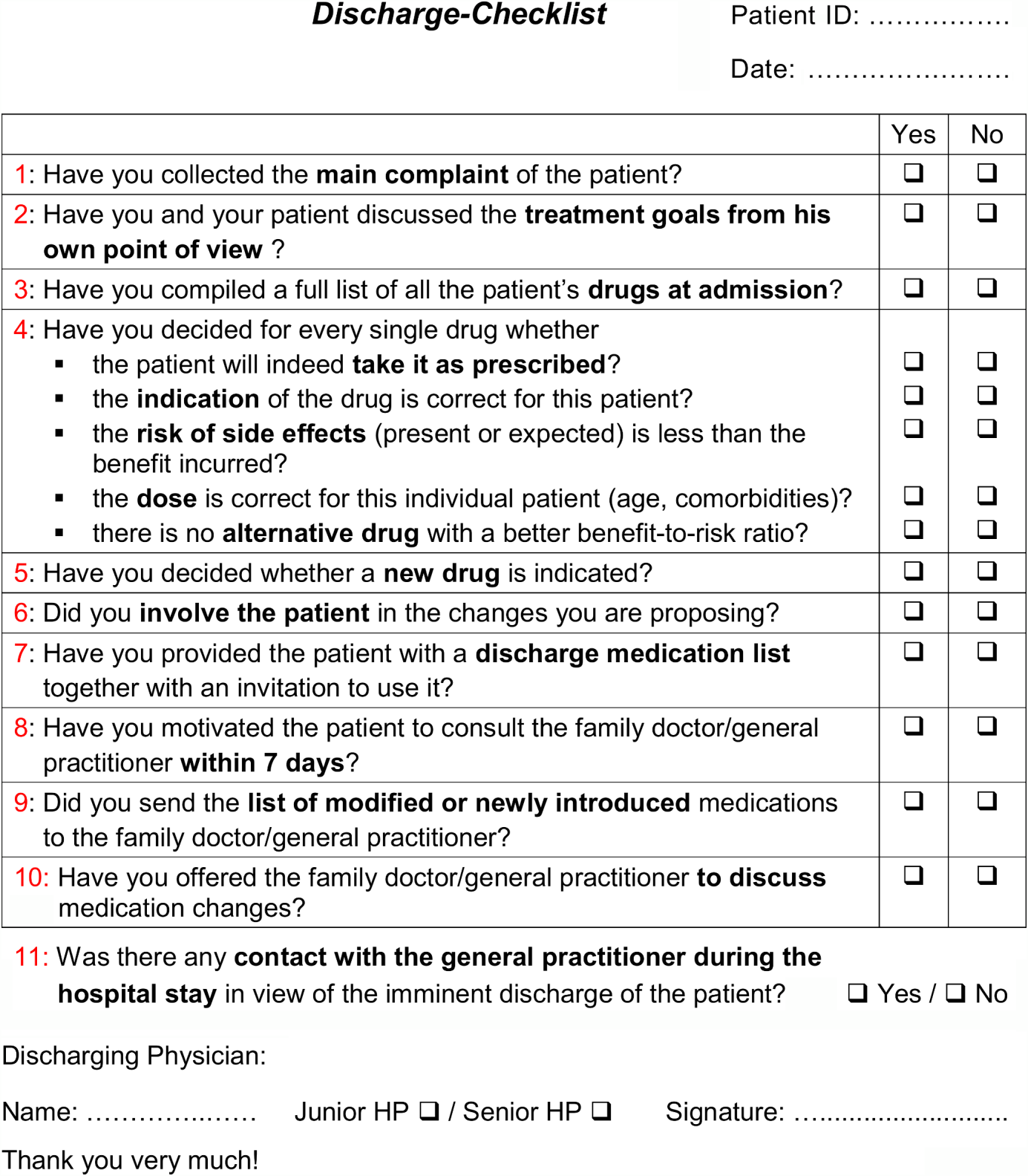


**Invitation to discuss medication changes** (in bold at the end of the discharge letters to PCPs)

Dear general practitioner

We strive to improve the discharge management of our patients. We are happy to discuss medication changes with you. Please call us at

0xx xxx xx xx (name, function) or 0xx xxx xx xx (name, function).

Thank you!

**eFigure 2. Kaplan-Meier Plot of Emergency Department (ED) Visits**


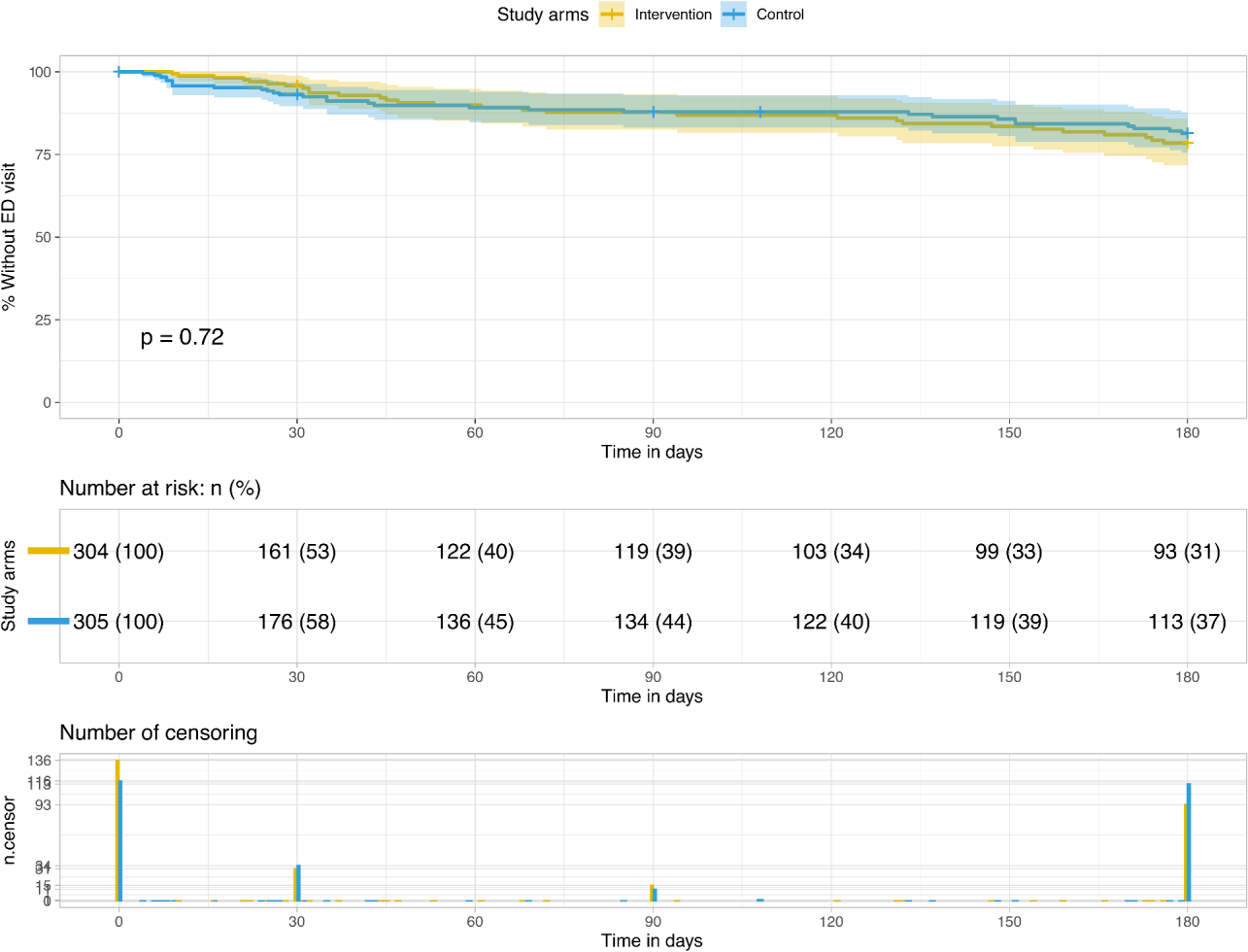


**eFigure 3. Kaplan-Meier Plot of Other Medical Consultations**


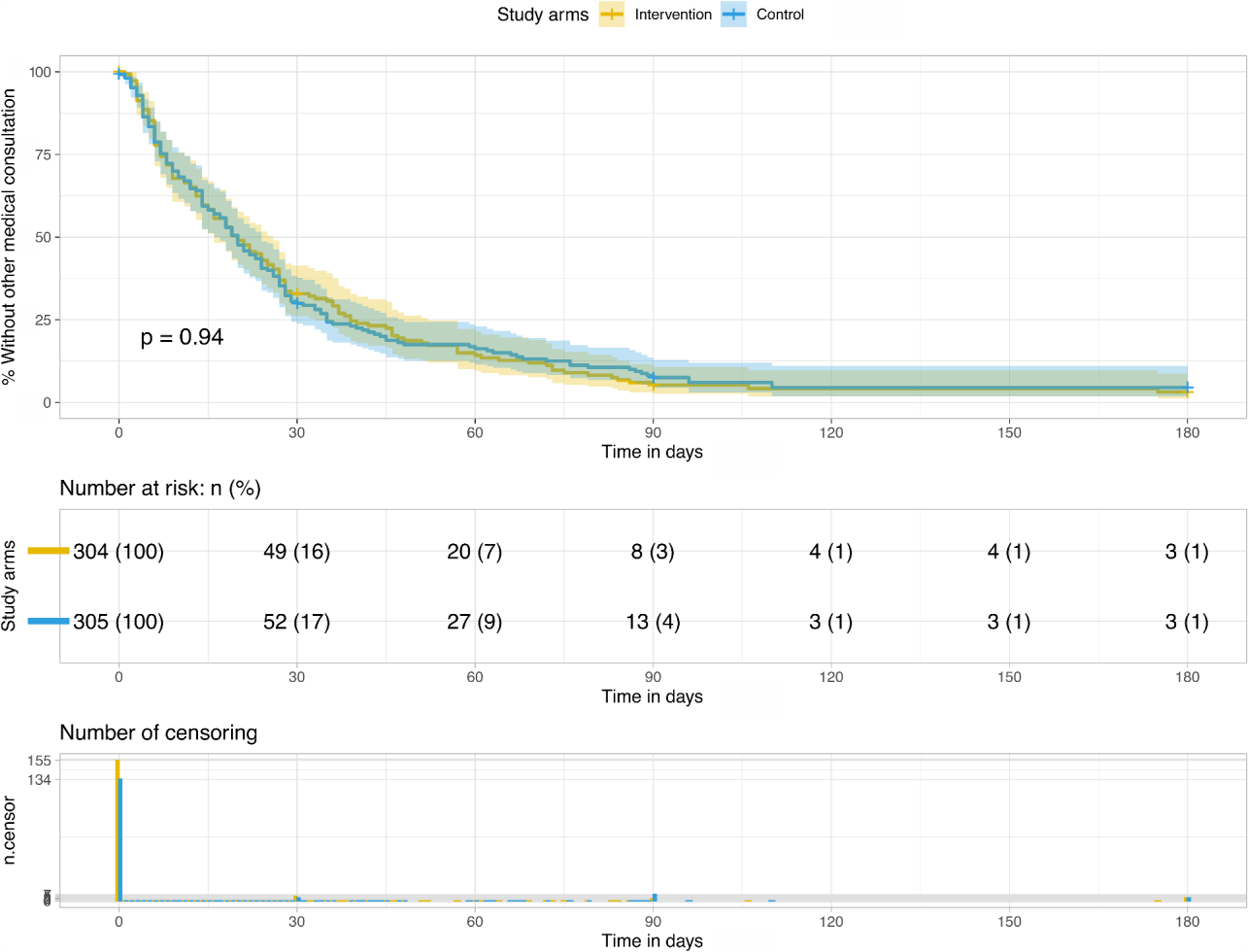


**eFigure 4. ATC Therapeutic Subgroups at Discharge**


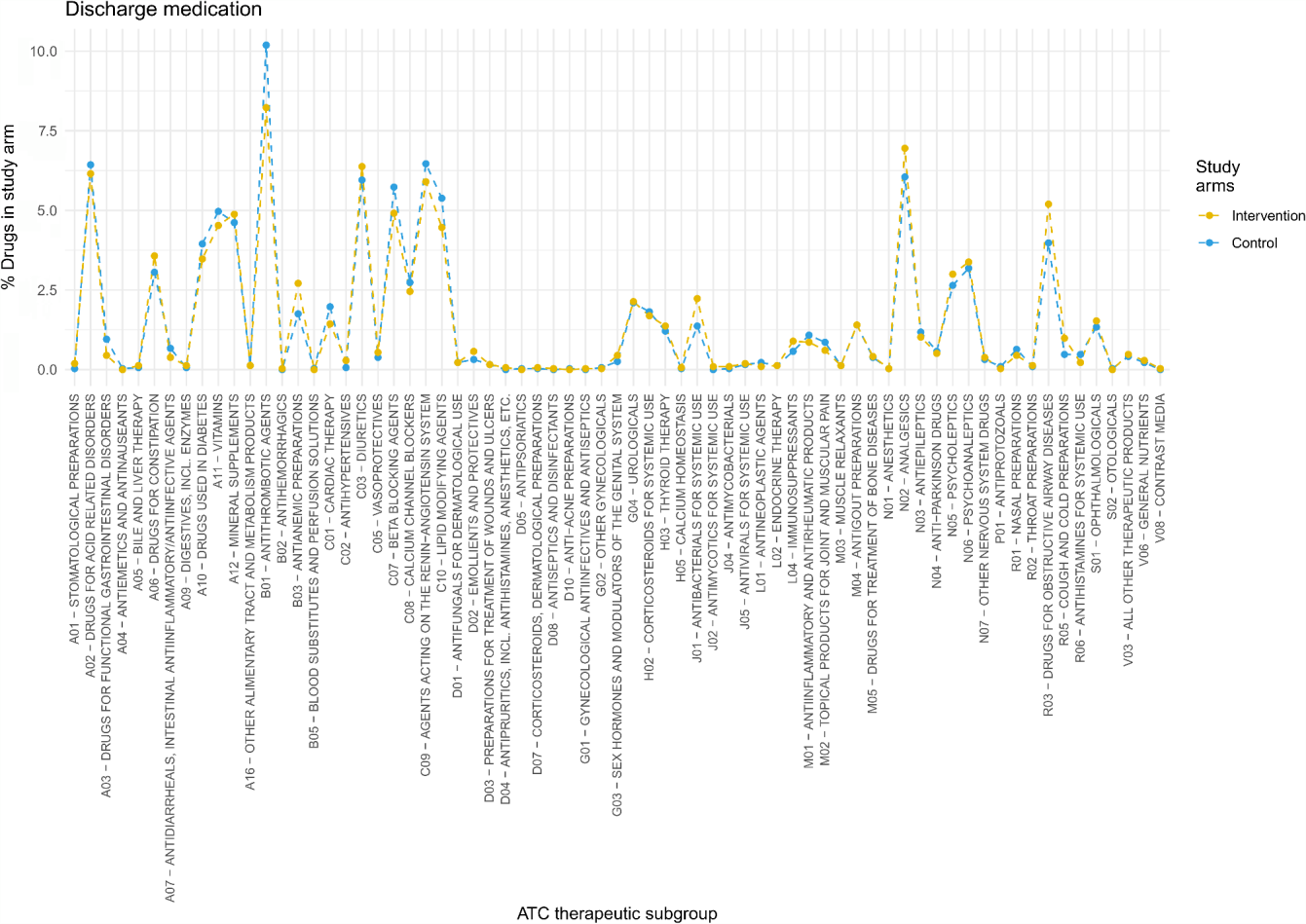


**eFigure 5. ATC Pharmacological Subgroups at Discharge**


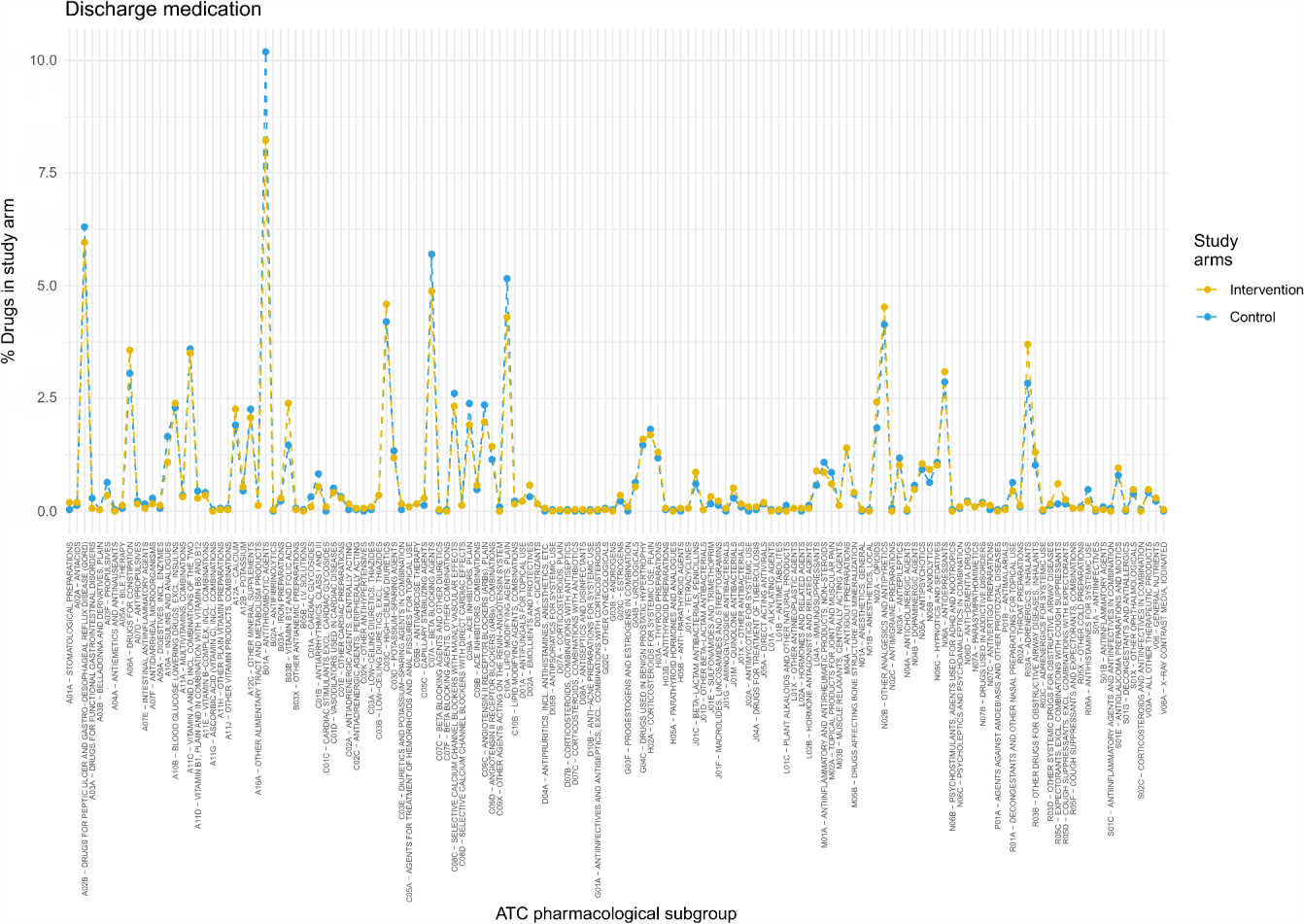


**eReferences**

1. Grischott T. The Shiny Balancer - Software and imbalance criteria for optimally balanced treatment allocation in small RCTs and cRCTs. BMC Med Res Methodol. 2018;18(108). doi:10.1186/s12874-018-0551-5

2. van Buuren S, Groothuis-Oudshoorn K. mice: Multivariate Imputation by Chained Equations in R. Journal of Statistical Software. 2011;45(3):1-67.

3. Robitzsch A, Grund S. miceadds: Some Additional Multiple Imputation Functions, Especially for 'mice'. 2021.

4. Grischott T, Zechmann S, Rachamin Y, et al. Improving inappropriate medication and information transfer at hospital discharge: study protocol for a cluster RCT. Implement Sci. 2018;13(1):155. doi:10.1186/s13012-018-0839-1

5. Freedman LS. Tables of the number of patients required in clinical trials using the logrank test. Stat Med. 1982;1(2):121-9. doi:10.1002/sim.4780010204

6. Xie T, Waksman J. Design and sample size estimation in clinical trials with clustered survival times as the primary endpoint. Stat Med. 2003;22(18):2835-46. doi:10.1002/sim.1536

7. Austin PC. A Tutorial on Multilevel Survival Analysis: Methods, Models and Applications. International statistical review = Revue internationale de statistique. 2017;85(2):185-203. doi:10.1111/insr.12214
