# Supplementary material for: Medication Review and Enhanced Information Transfer at Discharge of Older Patients with Polypharmacy: a Cluster-Randomized Controlled Trial in Swiss Hospitals
Source: J Gen Intern Med. 2022 Aug 31;38(3):610–8. doi: 10.1007/s11606-022-07728-6 (PMC9432794; doi:10.1007/s11606-022-07728-6)
Supplement: Supplementary file 2 — (PDF 1.22 mb) [file 11606_2022_7728_MOESM2_ESM.pdf]

Clinical Study Protocol

Improving Inappropriate Medication and Information Transfer at Hospital Discharge. A Cluster-RCT

HDS – The Hospital Discharge Study

**Study Type:**	Health-related intervention
**Study Categorization:**	Other Clinical Trial Category A
**Study Registration:**	Study registry: ISRCTN<br>Registration number: ISRCTN18427377
**Study Identifier:**	BASEC-No. 2018-00215<br>NRP 74 Project 19
**Sponsor-Investigator and Principal Investigator:**	Dr. med. Stefan Neuner-Jehle MPH<br>Institute of Primary Care<br>UniversityHospital Zurich<br>Pestalozzistrasse 24<br>CH-8091 Zurich<br>Phone: +41 (0) 44 255 98 55<br>Fax: +41 (0) 44 255 90 97<br>Email: stefan.neuner-jehle@usz.ch
**Study Intervention:**	Teaching session
**Protocol Version and Date:**	Version 1.1 of March 07, 2018<br>Replaces version 1.0 from January 08, 2018

CONFIDENTIAL

The information contained in this document is confidential and the property of the Institute of Primary Care of the UniversityHospital Zurich. The information may not – in full or in part – be transmitted, reproduced, published, or disclosed to others than the applicable Independent Ethics Committee(s) and Competent Authority(ies) without prior written authorization from the Institute of Primary Care of the UniversityHospital Zurich, except to the extent necessary to obtain informed consent from those participants who will participate in the study.

SIGNATURE PAGES

**Study number**	Study registry: ISRCTN<br>Registration number: ISRCTN18427377
**Study Title**	Improving Inappropriate Medication and Information Transfer at Hospital Discharge.<br>A Cluster-RCT

Sponsor-Investigator and Principal Investigator:

This clinical trial protocol was subject to critical review and has been approved by the Sponsor-Investigator. The information herein is consistent with

- the current risk/benefit evaluation of the intervention,
- the moral, ethical and scientific principles governing clinical research as set out in the current version of the Declaration of Helsinki, Good Clinical Practice.

Dr. med. Stefan Neuner-Jehle MPH

Zurich, 5.4.18

Place/Date

Signature

Local Investigator at Site Bern:

- Prof. Dr. med. Nicolas Rodondi

Bern, 27.3.2018

Place/Date

Signature

Collaborators:

- Prof. Dr. med. Thomas Rosemann PhD

Zurich, 27.3.2018

Place/Date

Signature

- Prof. Dr. med. Oliver Senn MPH

Zurich, 4.4.18

Place/Date

Signature

- PD Dr. med. Corinne Chmiel

Zurich, 4.4.18

Place/Date

Signature

- Dr. med. Stefan Zechmann

Zurich, 4.4.18

Place/Date

Signature

- Dr. med. Stefan Markun

Zurich, 4.4.2018

Place/Date

Signature

- Dr. med. Thomas Grischott MSc

Zurich, 4.4.2018

Place/Date

Th. Grischott

Signature

Biometrician:

Dr. med. Thomas Grischott MSc

Zurich, 4.4.2018

Place/Date

Th. Grischott

Signature

TABLE OF CONTENTS

SIGNATURE PAGES 2
STUDY SYNOPSIS 7
LIST OF ABBREVIATIONS 11
STUDY SCHEDULE 12
1 INVESTIGATORS AND STUDY ADMINISTRATIVE STRUCTURE 13
1.1 Sponsor-Investigator and Principal Investigator 13
1.2 Other Investigators 13
1.3 Statistician (Biometrician) 13
1.4 Monitoring Institution 13
2 ETHICAL AND REGULATORY ASPECTS 14
2.1 Study Registration 14
2.2 Categorization of the Study 14
2.3 Competent Ethics Committee (CEC) 14
2.4 Ethical Conduct of the Study 14
2.5 Declaration of Interest 15
2.6 Participant Information and Informed Consent 15
2.7 Participant Privacy and Confidentiality 15
2.8 Early Termination of the Study 16
2.9 Protocol Amendments 16
3 INTRODUCTION 17
3.1 Background and Rationale 17
3.2 Study Intervention and Indication 17
3.3 Clinical Evidence to Date 18
3.4 Justification of Study Intervention 18
3.5 Explanation for Choice of Comparator Intervention 19
3.6 Risks and Benefits 19
3.7 Study Population 20
4 STUDY OBJECTIVES 21
4.1 Overall Objective 21
4.2 Primary Objective 21
4.3 Secondary Objectives 21
4.4 Safety Objectives 21
5 STUDY OUTCOMES 22
5.1 Primary Outcome 22
5.2 Secondary Outcomes 22
5.3 Process Evaluation Outcomes 23
6 STUDY DESIGN AND COURSE OF STUDY 24
6.1 General Study Design and Justification of the Design 24
6.2 Study Duration and Study Schedule 25
6.3 Methods of Minimizing Bias 25
6.3.1 Randomization 26
6.3.2 Blinding Procedures 26

6.4 Unblinding Procedures (Code break) 26
7 STUDY POPULATION 27
7.1 Eligibility Criteria 27
7.1.1 Inclusion Criteria 27
7.1.2 Exclusion Criteria 27
7.2 Recruitment and Screening 27
7.3 Assignment to Study Groups 28
7.4 Criteria for Withdrawal/Discontinuation of Participants 28
8 STUDY INTERVENTION 29
8.1 General Information and Administration of Interventions 29
8.1.1 Study Intervention 29
8.1.2 Control Intervention 30
8.2 Compliance with Intervention 30
8.3 Data Collection and Follow-up for Withdrawn Participants 30
8.4 Concomitant Interventions 30
9 STUDY PROCEDURES 31
9.1 Study Flow Chart/Table of Study Procedures and Assessments 31
9.2 Assessments of Outcomes 32
9.2.1 Assessment of Primary Outcome 32
9.2.2 Assessment of Secondary Outcomes 33
9.2.3 Assessment of Covariates 33
9.2.4 Assessment of Safety Outcomes 33
9.2.5 Assessments in Participants who Prematurely Stop the Study 33
9.3 Procedures at Each Study Stage 33
9.3.1 R: Recruitment of HPs 33
9.3.2 T: Teaching of HPs 33
9.3.3 A: Patient Admission 33
9.3.4 D = T0: Patient Discharge (Baseline) 34
9.3.5 T1: 1 Month After Discharge 34
9.3.6 T3: 3 Months After Discharge 34
9.3.7 T6: 6 Months After Discharge 34
10 SAFETY 35
10.1 Definition of Serious Adverse Events 35
10.2 Recording and Assessment of Serious Adverse Events 35
10.3 Reporting of Serious Adverse Events 35
10.4 Follow up of (Serious) Adverse Events 35
10.5 Urgent Safety Measures 36
10.6 Early Termination of the Study due to Safety Concerns 36
11 STATISTICAL METHODS 37
11.1 Hypothesis 37
11.2 Determination of Sample Size 37
11.3 Planned Analyses 37
11.3.1 Datasets to be Analyzed, Analysis Populations 37
11.3.2 Primary Analysis 37

11.3.3 Secondary Analyses ... *38*
11.3.4 Interim Analysis ... *38*
11.3.5 Deviations from the Original Statistical Plan ... *38*
11.4 Handling of Missing Data and Dropouts ... 38
12 ELIGIBILITY OF THE PROJECT SITES ... 39
13 DATA QUALITY ASSURANCE AND CONTROL ... 40
13.1 DATA HANDLING AND RECORD KEEPING ... 40
13.1.1 Case Report Forms ... *40*
13.1.2 Specification of Source Documents ... *40*
13.1.3 Record Keeping/Archiving ... *41*
13.2 Data Management ... 41
13.3 Routine Monitoring ... 41
13.4 Audits and Inspections ... 41
13.5 Confidentiality, Data Protection ... 42
14 PUBLICATION AND DISSEMINATION POLICY ... 43
15 FUNDING AND SUPPORT ... 44
16 INSURANCE ... 45
17 REFERENCES ... 46
18 APPENDICES ... 50
18.1 List of Study Instruments ... 50

STUDY SYNOPSIS

**Sponsor-Investigator:**	Dr. med. Neuner-Jehle Stefan MPH
**Study Title:**	Improving Inappropriate Medication and Information Transfer at Hospital Discharge.<br>A Cluster-RCT
**Short Title/Study ID:**	HDS – The Hospital Discharge Study
**Protocol Version and Date:**	Version 1.1 of March 07, 2018
**Trial registration:**	Study registry: ISRCTN<br>Registration number: ISRCTN18427377
**Study category and Rationale:**	Other clinical study, category A<br>The intervention will comprise aspects of communication as well as critical reviews and optimizations of discharge medication plans with the possibility to immediately reverse any change in case of unintended side-effects.
**Background and Rationale:**	Inappropriate medication and polypharmacy increase morbidity, hospitalization rate, costs and mortality in multimorbid patients. At discharge from hospital, polypharmacy is substantially increased compared to admission. Although interventions to reduce polypharmacy exist in variable grades of complexity and dissemination, up to now their use has been rather restricted to specialized settings like geriatric medicine or pharmacology but not widely adopted at the crucial interface between hospital physicians (HPs) and general practitioners (GPs).<br>Moreover, optimally reduced medication plans at hospital discharge do not suffice to achieve sustained polypharmacy reduction. Switching back to the pre-hospitalization medication scheme is known to be a common pitfall to be avoided on the way to reduce polypharmacy. Therefore, a consensus between HPs and referring GPs with regard to the discharge medication plans, resulting from the involvement of the latter in the prescription/deprescription decisions at hospital discharge, is a suitable measure to increase adoption rates of optimized discharge medication plans.<br>The present trial falls in line with other recent studies which showed that planned discharge interventions can considerably reduce re-hospitalization rates and extend time to readmission. To our knowledge, it will be the first study to analyze the combination of a brief deprescribing intervention with a standardized communication strategy between HPs and GPs, at discharge from hospital and in the early post-discharge period.

**Objectives:**	To test the hypothesis that a simple medication review tool in combination with a defined communication strategy at hospital discharge leads to prolonged hospital re-admission times compared to usual care (control group), and has the potential to improve patients' health outcomes.<br><br>To identify best practice of how to implement this approach in daily routine at hospital discharge.<br><br>To estimate the potential cost savings of this approach.<br><br>To foster dissemination of this approach (if successful) among Swiss hospital wards and GPs.
**Outcomes:**	Primary outcome:<br>• Time (days) without readmission to hospital, within 6 months<br><br>Secondary outcomes:<br>• Hospital readmission rates within 1, 3 and 6 months<br>• Number of emergency department (ED) visits within 1, 3 and 6 months<br>• Number of GP encounters within 1, 3 and 6 months<br>• Death during follow-up of 6 months<br>• Differences in the number of drugs per patient at discharge, 1, 3 and 6 months, between intervention and control group.<br>• Anatomical therapeutic chemical classes (ATC codes) of the drugs prescribed/de-prescribed<br>• Proportion of potentially inappropriate medications (PIMs) at discharge, 1, 3 and 6 months<br>• Patients' quality of life at discharge, 1, 3 and 6 months<br>• Frequency of utilization of the communication offer (phone calls of GPs to HPs)<br>• Ratings of feasibility/acceptance by HPs<br>• Barriers and enablers of deprescribing among HPs and among patients<br>• Dropout rates of HPs, GPs and patients
**Study Design:**	Single-center double-blind cluster-randomized parallel-controlled clinical trial with a 6-month follow up.
**Inclusion/Exclusion Criteria:**	Inclusion criteria: In-hospital patients will be eligible if they are 60 years or older and prescribed 5 or more drugs.<br><br>Exclusion criteria: Cognitive inability to follow study procedures or end-stage disease with a life expectancy of less than 3 months.

**Study Intervention:**	1: A “teach-the teacher” training session for the senior HPs in charge of postgraduate education and supervision of the interns (assistant physicians). The purpose of this training of 2 hours duration is to integrate the discharge procedure (see below) into the daily work of the intern HPs during the study period. Consecutively, the senior HPs will teach the interns how to apply the structured discharge procedure. This will assure the consistency of the intervention, as the fluctuation among interns is high due to job rotations within hospitals.<br><br>2: The intern HP performs a critical review of the medication plan, supervised by his senior HP, discusses the results of this review and his suggestions with the patient, and creates a medication plan.<br><br>3: This revised medication plan is communicated to the patient’s GP with an invitation for discussion.
**Reference Intervention:**	The HPs in the control group will undergo a 2-hour educative session addressing multimorbidity, patient in- and exclusion and the handling of the different data collection forms.
**Number of Participants with Rationale:**	Based on published readmission rates, an exponential survival curve has been modeled. We defined a delay in time to readmission of 25% as relevant. This translates into a difference in medians of 23 days (from 93 to 116 days) and corresponds to a hazard ratio of 0.80 between intervention and control group participants. We assumed a two-sided alpha of 0.05, a power of 80%, an intraclass correlation coefficient (ICC) of 0.02, an overall censoring probability of 40%, and a cluster size of 50 patients per senior HP. Thus, 21 senior HPs and 1’050 patients per arm are needed, i.e. 42 senior HPs and 2’100 patients in total.<br><br>An interim analysis with 50% of the full sample size is planned in case of poor recruitment success.
**Study Duration:**	Patients will be recruited during 4 months or until the designated number of patients per cluster is reached, and follow up will last for 6 months for each patient.
**Study Schedule:**	Pilot test, first participant in (planned): 03/2018<br>RCT, last participant out (planned): 03/2019

**Investigators:**	• Dr. Stefan Neuner-Jehle, Institute of Primary Care, UniversityHospital Zurich, CH-8091 Zurich<br>Tel. 044 255 98 55, stefan.neuner-jehle@usz.ch<br>• Prof. Nicolas Rodondi, Institute of Primary Health Care, University Hospital Bern, CH-3012 Bern<br>Tel. 031 631 57 93, nicolas.rodondi@biham.unibe.ch<br>• Prof. Thomas Rosemann, Institute of Primary Care, UniversityHospital Zurich, CH-8091 Zurich<br>Tel. 044 255 98 55, thomas.rosemann@usz.ch<br>• Prof. Oliver Senn, Institute of Primary Care, UniversityHospital Zurich, CH-8091 Zurich<br>Tel. 044 255 98 55, oliver.senn@usz.ch<br>• PD Corinne Chmiel, Institute of Primary Care, UniversityHospital Zurich, CH-8091 Zurich<br>Tel. 044 255 98 55, corinne.chmiel@usz.ch<br>• Dr. Stefan Zechmann, Institute of Primary Care, UniversityHospital Zurich, CH-8091 Zurich<br>Tel. 044 255 98 55, stefan.zechmann@usz.ch<br>• Dr. Stefan Markun, Institute of Primary Care, UniversityHospital Zurich, CH-8091 Zurich<br>Tel. 044 255 98 55, stefan.markun@usz.ch<br>• Dr. Thomas Grischott, Institute of Primary Care, UniversityHospital Zurich, CH-8091 Zurich<br>Tel. 044 255 98 55, thomas.grischott@usz.ch
**Study Center:**	Institute of Primary Care, University of Zurich
**Statistical Considerations:**	Pilot test: Mixed methods approach to achieve quantitative (e.g. ratings on a 5-point Likert scale) as well as qualitative (e.g. awareness, perception) results.<br><br>RCT: Intent-to-treat (ITT) approach. Descriptive statistics to describe the study population. Baseline characteristics will be compared between control and intervention group using *t*-test, Mann-Whitney *U* test, χ^2-test or Fisher's test, according to the type of data. The primary outcomes will be compared using Kaplan-Meier and log rank tests. To assess factors which may affect readmission, Cox regression will be used considering senior HPs as clusters. Determinants associated with a change of the medication will be investigated by exploratory, multivariate regression analysis. For secondary outcomes, parametric (*t*-tests) or non-parametric tests (χ^2- and *U* tests) will be used where appropriate.
**GCP Statement:**	This study will be conducted in compliance with the protocol, the current version of the Declaration of Helsinki, the ICH-GCP as well as all national legal and regulatory requirements.

LIST OF ABBREVIATIONS

AE	Adverse Event
ASR	Annual Safety Report
ATC	Anatomical Therapeutic Chemical Classification System
CEC	Competent Ethics Committee
CI	Confidence Interval
ClinO	Clinical Trial Ordinance (Verordnung über klinische Versuche)
(e)CRF	(Electronic) Case Report Form
ED	Emergency Department
GCP	Good Clinical Practice
GP	General Practitioner
HFG	Bundesgesetz über die Forschung am Menschen (Humanforschungsgesetz)
HP	Hospital Physician
ICH	International Council for Harmonisation<br>of Technical Requirements for Pharmaceuticals for Human Use
ISRCTN	International Standard Registered Clinical/soCial sTudy Number
NRP 74	National Research Programme "Smarter Health Care"
OPERAM	OPtimising thERapy to prevent Avoidable hospital admissions<br>in the Multimorbid elderly
PI	Principal Investigator
PIM	Potentially Inappropriate Medication
QoL	Quality of Life
(c)RCT	(Cluster-)Randomized Controlled Trial
RR	Relative Risk
SAE	Serious Adverse Event
SAMW	Schweizerische Akademie der Medizinischen Wissenschaften
SNCTP	Swiss National Clinical Trial Portal
SOP	Standard Operating Procedure
TMF	Trial Master File
ZGB	Schweizerisches Zivilgesetzbuch

STUDY SCHEDULE

Study Period	HP Recruitment and Teaching		Hospital Stay		Post Hospital Follow Up			Eval. and Dissem.
Time Intervall	01/2018-06/2018		05/2018-03/2019					10/2018 -12/2020
Point in Time (Months after Discharge)				0	1	3	6	
**Study Stage**	**R**	**T**	**A**	**D = T0**	**T1**	**T3**	**T6**	
HP Recruitment	x							
HP Information and Informed Consent	x							
Clinc Characteristics	x							
Randomization	x							
HP Teaching		x						
Patient Selection and In-/Exclusion			x					
Patient Information and Informed Consent			x					
Biometric Patient Data			x					
Medication Plan				x				
Changes in Medication				x				
Readmission with Date (if applicable)					x	x	x	
ED Visits/GP Encounters					x	x	x	
ATC-Codes				x				
Number of Drugs				x	x	x	x	
Quality of Life				x	x	x	x	
Frequency of GP-HP Communication					x			
Time for Applying Intervention				x				
Dropout Rates					x	x	x	
Feasibility/Acceptance and Barriers/Enablers								x
Debriefing of Control HPs								x
Data Analysis								x
Final Reporting/ Publication/Dissemination								x

Study stages: R = Recruitment, T = Teaching, A = Admission, D = T0 = Discharge, T1/2/3 = 1/2/3 months after discharge

1 INVESTIGATORS AND STUDY ADMINISTRATIVE STRUCTURE

1.1 Sponsor-Investigator and Principal Investigator

Dr. med. Neuner-Jehle Stefan MPH

Institute of Primary Care, UniversityHospital Zurich, Pestalozzistrasse 24, CH-8091 Zurich
Tel. 044 255 98 55, Fax 044 255 90 97, Email stefan.neuner-jehle@usz.ch

1.2 Other Investigators

Prof. Dr. med. Nicolas Rodondi

Institute of Primary Health Care, University Hospital Bern, Gesellschaftsstr. 49, CH-3012 Bern
Tel. 031 631 57 93, Fax 031 631 58 71, Email nicolas.rodondi@biham.unibe.ch

Prof. Dr. med. Thomas Rosemann PhD

Institute of Primary Care, UniversityHospital Zurich, Pestalozzistrasse 24, CH-8091 Zurich
Tel. 044 255 98 55, Fax 044 255 90 97, Email thomas.rosemann@usz.ch

Prof. Dr. med. Oliver Senn MPH

Institute of Primary Care, UniversityHospital Zurich, Pestalozzistrasse 24, CH-8091 Zurich
Tel. 044 255 98 55, Fax 044 255 90 97, Email oliver.senn@usz.ch

PD Dr. med. Corinne Chmiel

Institute of Primary Care, UniversityHospital Zurich, Pestalozzistrasse 24, CH-8091 Zurich
Tel. 044 255 98 55, Fax 044 255 90 97, Email corinne.chmiel@usz.ch

Dr. med. Stefan Zechmann

Institute of Primary Care, UniversityHospital Zurich, Pestalozzistrasse 24, CH-8091 Zurich
Tel. 044 255 98 55, Fax 044 255 90 97, Email stefan.zechmann@usz.ch

Dr. med. Stefan Markun

Institute of Primary Care, UniversityHospital Zurich, Pestalozzistrasse 24, CH-8091 Zurich
Tel. 044 255 98 55, Fax 044 255 90 97, Email stefan.markun@usz.ch

Dr. med. Thomas Grischott MSc

Institute of Primary Care, UniversityHospital Zurich, Pestalozzistrasse 24, CH-8091 Zurich
Tel. 044 255 98 55, Fax 044 255 90 97, Email thomas.grischott@usz.ch

1.3 Statistician (Biometrician)

Dr. med. Thomas Grischott MSc

Institute of Primary Care, UniversityHospital Zurich, Pestalozzistrasse 24, CH-8091 Zurich
Tel. 044 255 98 55, Fax 044 255 90 97, Email thomas.grischott@usz.ch

1.4 Monitoring Institution

Institute of Primary Care, UniversityHospital Zurich, Pestalozzistrasse 24, CH-8091 Zurich,
Tel. 044 255 98 55, Fax 044 255 90 97

2 ETHICAL AND REGULATORY ASPECTS

Before this study will be conducted, the protocol, the proposed participant information and consent form as well as other study-specific documents will be submitted to a properly constituted Competent Ethics Committee (CEC) in agreement with local legal requirements, for formal approval.

The decision of the CEC concerning the conduct of the study will be made in writing to the Sponsor-Investigator before commencement of this study. The clinical study will only begin once approval from the CEC has been received.

2.1 Study Registration

The study has been registered with the ISRCTN primary clinical trial registry (www.isrctn.com) for studies designed to assess the efficacy of health interventions in a human population, and with the Swiss Federal Complementary Database SNCTP (www.kofam.ch).

2.2 Categorization of the Study

Other Clinical Trial Category A

The health-related study intervention entails only minor risks and minimal burdens for the medical personnel and patients involved. The former will be subjected to educative training sessions, and on the patient level the intervention will involve a critical review and potential optimization of discharge medication plans by trained physicians with the possibility to immediately reverse any change in case of unintended side-effects.

2.3 Competent Ethics Committee (CEC)

Approval from the appropriate constituted Competent Ethics Committee is sought for the clinical trial. The reporting duties and allowed timeframe will be respected. No substantial amendments will be made to the protocol without prior CEC approval, except where necessary to eliminate apparent immediate hazards to study participants. Premature study end or interruption of the study is reported within 15 days. The regular end of the study will be reported to the CEC within 90 days, the final study report or publication shall be submitted within one year after study end. Amendments are reported according to chapter 2.9.

2.4 Ethical Conduct of the Study

The study will be carried out in accordance with principles enunciated in the current version of the Declaration of Helsinki, the guidelines of Good Clinical Practice (GCP) issued by the International Council for Harmonisation (ICH), and Swiss competent authority's requirements.

The CEC will receive annual safety and interim reports and be informed about non-substantial amendments, the course of the study, and the study stop/end in agreement with local requirements.

2.5 Declaration of Interest

The study team declares that no conflict of interest (independence, intellectual, financial, proprietary etc.) exists.

2.6 Participant Information and Informed Consent

The investigator must explain to each participant the nature of the study, its purpose, the procedures involved, the expected duration, the potential risks and benefits and any discomfort it may entail. Each participant will be informed that the participation in the study is voluntary and that he/she may withdraw from the study at any time and that withdrawal of consent will not affect his/her subsequent medical treatment.

The participant will be informed that his/her medical records may be examined by authorized individuals other than their treating physician.

All participants for this study will be provided a participant information sheet and a consent form describing this study and providing sufficient information for participants to make an informed decision about their participation in this study.

The participant information sheet and the consent form are submitted with the protocol for review and approval by the CEC (see appendix). The formal consent of a participant, using the approved consent form, will be obtained before that participant is submitted to any study procedure.

The participant should read and consider the statement before signing and dating the informed consent form, and will be given a copy of the signed document. The consent form will also be signed and dated by the investigator (or his designee) and it will be retained as part of the study records.

2.7 Participant Privacy and Confidentiality

The investigators are liable to treat the entire information related to the study and the compiled data strictly confidentially. Anonymity is warranted in publications and public presentations. Any passing-on of information to persons that are not directly involved in the study must be approved by the owner of the information.

Data generation, transmission, archiving and analysis of personal data within this study strictly follow the current Swiss legal requirements for data protection. Prerequisite is the voluntary approval of the participant given by signing the informed consent prior start of participation of the clinical trial.

Individual participant medical information obtained as a result of this study is considered confidential and disclosure to third parties is prohibited. The participants' confidentiality will be further ensured by utilizing participant identification code numbers to correspond to treatment data in the computer files.

Such medical information may be given to the participant's personal physician or to other appropriate medical personnel responsible for the participant's welfare, if the patient has given his/her written consent to do so.

Data generated as a result of this study will be available for inspection on request by the monitors and by the CEC.

2.8 Early Termination of the Study

The Sponsor-Investigator and competent authority may discontinue the study prematurely according to the following circumstances:

- Ethical concerns
- Insufficient participant recruitment
- When the safety of the participants is doubtful or at risk, respectively
- Alterations in accepted clinical practice that make the continuation of a clinical trial unwise
- Early evidence of benefit or harm of the experimental intervention

2.9 Protocol Amendments

No substantial amendments (significant changes) will be implemented without prior approval of the CEC.

Significant changes to be authorized by the CEC are the following:

- Changes affecting the participants' safety and health, or their rights and obligations
- Changes to the protocol, and in particular changes based on new scientific knowledge which concern the trial design, the method of investigation, the endpoints or the form of statistical analysis
- A change of trial site, or conducting the clinical trial at an additional site, or
- A change of sponsor, coordinating investigator or investigator responsible at a trial site

Under emergency circumstances, deviations from the protocol to protect the rights, safety and well-being of human participants may proceed without prior approval of the sponsor and the CEC. Such deviations shall be documented and reported to the sponsor and the CEC as soon as possible.

All non-substantial amendments will be communicated to the CEC within the Annual Safety Report (ASR).

3 INTRODUCTION

3.1 Background and Rationale

a. The polypharmacy issue

Inappropriate medication and polypharmacy increase morbidity, hospitalization rates, costs and mortality in multimorbid patients (10, 22). At discharge from hospital, the prevalence of polypharmacy is dramatically higher than at hospital admission. The interface between hospital physicians (HPs) and general practitioners (GPs) is of utmost relevance for the reduction of polypharmacy and drug-related side effects (33, 40).

b. The hospital discharge issue

Since appropriate communication and exchange of information between hospital staff and community caregivers at discharge from hospital is crucial for a successful health care system, there is significant room for improvement (4, 17, 20, 21, 23, 41, 53). Switching back to pre-hospitalization medication scheme after hospitalization has been identified as a major problem for failing to reduce polypharmacy in the long term (2). Face-to-face interaction or collaborative agreements between hospital pharmacists and GPs lead to higher adoption rates of discharge medication plans compared to one-way communication approaches (36).

c. Synthesis

To our knowledge, this trial will be the first to test the combination of a brief deprescribing intervention and a standardized communication strategy between HPs and GPs at discharge from hospital and in the early post-discharge period. By recording time to readmission (within 6 months) and readmission rates after 1, 3 and 6 months, we aim to show that this combined strategy has the potential to prolong the former and decrease the latter of these outcomes in addition to reducing the mere number of drugs prescribed.

Additionally, knowledge about barriers and facilitators for the implementation of approaches like ours in real-life settings is crucial for success and dissemination. The existing literature is mainly limited to GPs' and emergency departments' settings (7, 37, 42), but knowledge about barriers and enhancers towards deprescribing in the transition care setting is scarce. This gives the rationale to explore these factors by qualitative and mixed-methods research in order to provide recommendations for dissemination.

3.2 Study Intervention and Indication

The intervention takes place on different levels with different target populations:

a. Teaching session for the senior HP in charge of postgraduate training and supervision of the interns (assistant physicians)

The purpose of this training of two hours duration is to integrate the discharge procedure (see below) into the daily work of the HPs during the study period. Consecutively, the senior HPs will teach the interns how to apply the structured discharge procedure. This will guarantee the consistency of the intervention even if the fluctuation among interns is high due to organized job rotations within hospitals.

b. The intervention at discharge

The intern HP performs a critical review of the medication plan, supervised by his senior HP, discusses the results of this review and his suggestions with the patient and creates a discharge medication plan. Changes compared to the admission medication are communicated to the GP together with an offer to discuss these changes.

3.3 Clinical Evidence to Date

a. The polypharmacy issue

Interventions to reduce polypharmacy exist in variable grades of complexity, feasibility and dissemination (8, 9, 11, 14, 16, 35, 49). However, up to now their use is rather restricted to specialists (geriatricians, pharmacologists). In medical education the management of polypharmacy is of increasing importance and guidelines providing recommendations how to deal with polypharmacy are emerging (24, 34, 44). In a very recently published trial, a multifaceted approach with a web-based tool was effective in reducing potentially inappropriate prescribing (6).

The reduction of inappropriate medication is also the topic of several studies we carried out ourselves in the past years, focusing on the situation in Switzerland as well as abroad (27, 29, 38, 45).

In an ongoing cluster-randomized controlled study (cRCT), we use a 3 stepped procedure (15): a.) compilation of a comprehensive list of all drugs used by the patient; b.) systematic medication review, carefully considering indication, potential side effects, dosage, possible alternatives and whether a new drug might be indicated; and c.) discussion with the patient to identify her/his preferences, treatment aims and priorities. A pilot study has already shown that such an intervention is feasible in GPs' practices within 15 minutes, and that in this short consultation time, 9% of all drugs can be discontinued in mutimorbid patients with polypharmacy (32). First results of the main trial will be forthcoming soon.

b. The hospital discharge issue

Readmission after hospital discharge is a frequent event: In the US, one in 5 hospitalized Medicare beneficiaries is readmitted within 30 days, at a cost of more than $ 26 billion per year (19). In the recent years 2011 and 2012, these rates were quite stable, at 19% and 18.4%, respectively (12). In a German health assurance claims sample from 2003, the 30-day readmission rate among a sample of nearly 200’000 patients was very similar with 18.5% (47). Own (not published) data of the UniversityHospital of Zurich show a similar 30-day readmission rate of 17% and a 90-day readmission rate of 27% for multimorbid elderly patients (≥ 60 years).

Studies using different discharge interventions show effects between 15% and 50% readmission rate reduction, mainly at the 30-day post-discharge time point (28, 48). A recent systematic review from the Cochrane Collaboration (13) demonstrated an average 13% relative risk reduction for readmissions by planned discharge interventions (RR 0.87; 95% CI 0.79-0.97) from 15 trials, and time to readmission could be extended by half (from 12 to 18 days) in a frail population of older adults (≥60 years) by a transitional care program (46). Costantino et al. found that the earlier a multifaceted intervention took place after discharge from hospital the greater was the reduction in the readmission rate (7).

3.4 Justification of Study Intervention

In a cRCT in the primary care setting, we successfully pilot-tested the patient-centered drug review tool, which will be used in the planned study as a component of the discharge procedure, with regard to its practicability and feasibility (32). This pilot trial has been awarded the Swiss College of Primary Care Award in 2014. At present, we are conducting a cRCT that investigates the efficacy and safety of a medication review tool fostering deprescribing in the setting of GPs in northern and central Switzerland (15). This latter study has received a grant from the SAMW (Bangerter-Rhyner foundation) and will be carried out in collaboration with the Swiss OPERAM (34) team (PI: Prof. N. Rodondi, co-investigator of this project) who is currently developing a software to reduce inappropriate medication in the multimorbid elderly with polypharmacy across Europe. We think our project, focussing on communication about deprescribing in the transition

phase between hospital and ambulatory care, is complementary to the OPERAM design and fills a gap which is not addressed within the OPERAM project itself.

3.5 Explanation for Choice of Comparator Intervention

The control groups' senior HPs will undergo an educative session of the same duration as their peers in the intervention group but reduced in content, merely addressing multimorbidity and how to identify eligible patients according to the in- and exclusion criteria (first two items of the list in section 8.1.1). No modifications will be made to the established discharge procedures, so both the discharging interns and the further attending physicians will follow their usual transfer routines.

In the absence of a universally standardized discharge procedure, we opted for "usual care" in the above sense as our comparator method. If the study intervention successfully proves to have a beneficial effect on the chosen outcomes then the potential superiority of the intervention will be of considerable relevance to all adopters of the intervention.

3.6 Risks and Benefits

While taking a specific drug ideally has a beneficial effect on the health problem or risk factor targeted (e.g. reduction of the patient's risk of stroke) it may as well have a negative impact on other areas (e.g. adverse effects like hemorrhage, limitation of patient autonomy). In the case of polypharmacy among multimorbid patients, there is an increased risk of harmful effects on the patients' health due to the exponentially growing number of potential interactions and possible side effects (5, 9, 10, 14, 16, 22, 51).

Attempting to simultaneously adhere to both the ethical principles of care (beneficence) and precaution (non-maleficence) results in conflicting goals and requires careful risk-benefit considerations: In theory, discontinuing a drug with possible adverse effects may benefit the patient, but, on the other hand, it also means forgoing the therapeutic value of the drug with regard to the specific health condition targeted. Within the intervention of the present study, this assessment will be carried out deliberately for every single drug, and stopping a drug will only be suggested if, from a medical perspective, its (potential of already manifest) maleficent effects clearly exceed its beneficial potential. Our structured medication review process, based on a validated tool from geriatric medicine (11) and successfully tested in a pilot study (32) for an ongoing cRCT (15) encourages such considerations in the interest of patients.

Our goal is not to merely minimize the potential for harmful effects by reducing the number of drugs prescribed at hospital discharge but also to find an optimal balance between risk and benefit for the patients. To achieve this aim, our approach will favor the prioritization of indispensable and the identification of expendable medication, and the review of every single medicament together with the patient.

Of course, decisions within the deprescribing process have to be made together with the patients. The most pressing health complaints from the patients' view will guide all considerations from early stages within the medication review process. After being informed about their physicians' opinions and recommendations, the patients will then be able to decide about continuing or discontinuing any drug according to their own preferences, values and beliefs. Thus, the ethical principle of patient autonomy plays a key role in the study intervention.

Should symptoms of a previously controlled disease worsen after deprescribing, or if the activity of a disease increases in the post hospital course, then there will always be the option to revert the change and to re-introduce the original medication. In order to avoid virtually any pharmacology-related negative consequences of a deprescription, the discharging HP will be sensitized towards this problem (e.g. stepwise reduction of corticosteroids or anticoagulants).

Provided that our systematic approach will successfully prove its effectiveness in avoiding polypharmacy-induced health problems, we expect it to initiate a change in health professionals' and patients' thinking towards prioritizing, patient-centeredness, and refraining from the prescription of too many drugs for polymorbid patients. As a result, we hope to see a decrease in drug-caused health problems, medical complications, hospitalizations and unnecessary deaths, as well as a reduction in the costs entailed.

Our approach may serve as a model for an optimized discharge procedure, and we therefore expect that colleagues may welcome the suggestions in the dissemination phase. However, we also expect that a proportion of patients and caregivers may have difficulties to adopt this new kind of approach, as some barriers towards deprescribing are known from the literature (37, 42, 43, 52).

3.7 Study Population

An overwhelming proportion of multimorbid patients with extensive and complex medication regimes are elderly people who are taken care of by their GPs (39). Therefore, previous studies aimed at reducing polypharmacy – including one ongoing study by the authors of this proposal – restricted their scope to geriatric patients in the primary care setting (11, 14).

But an increased risk of introducing polypharmacy emerges also at the intersection of inpatient and outpatient medicine; at discharge from hospital the prevalence of polypharmacy is dramatically higher than at hospital admission (40, 50). We therefore chose hospital HPs responsible for the patients' discharge procedures and, in particular, for their discharge medication plans as the primary recipients of our study intervention. Hospitals of all types, levels and legal structures will be considered. Given the high fluctuation among interns between different wards of many hospitals and also between different hospitals, the educative training session will normally be offered to the senior HPs who, in turn, will teach the varying interns how to apply the structured discharge procedure.

The HPs will recruit the participants from inpatients according to the relatively non-restrictive in- and exclusion criteria (see section 7.1).

Switching back to pre-hospitalization medication schemes after hospitalization has been identified as a major barrier to a lasting reduction of polypharmacy (2). Therefore, we plan to involve the GPs in the optimized discharge process by offering them the opportunity to discuss the reduced medication schemes with the responsible HPs.

4 STUDY OBJECTIVES

4.1 Overall Objective

Overall objective of the cluster-randomized controlled study is to investigate means and consequences of reducing polypharmacy among multimorbid hospital patients as part of an improved discharge procedure. We thus hope to contribute to a reduction of potentially inappropriate medication and adverse drug effects and ultimately help patients to a prolonged time to hospital readmission.

4.2 Primary Objective

The primary objective of our project is to test the hypothesis that a simple medication review tool in combination with a defined communication strategy at hospital discharge leads to longer hospital readmission times compared to usual care (control group) and has the potential to improve the patients' health outcomes and quality of life (see secondary outcomes in section 5.2).

4.3 Secondary Objectives

Secondary objectives are:

- To identify best clinical practice of how to implement this approach in the daily routine at hospital discharge
- To estimate the potential of cost savings by this approach
- To foster dissemination of this approach (if successful) among Swiss hospital wards and GPs

4.4 Safety Objectives

Patient safety will be assessed by retrospectively, capturing all complications leading to GP encounters, ED visits and deaths that might possibly be related to modifications of the medication changes carried out within the study.

5 STUDY OUTCOMES

5.1 Primary Outcome

Outcome	Measurements
Time (days) without readmission<br>to hospital<br>within 6 months after discharge	Patient records tracking (patient questionnaires and/or calls, GP records, hospital records);<br>consecutive calculation

5.2 Secondary Outcomes

Outcomes	Measurements
Readmission rates<br>at 1, 3 and 6 months after discharge	Patient records tracking<br>(patient questionnaires and/or calls, GP records, hospital records)
Number of ED visits or GP encounters within 1, 3 and 6 months after discharge	Patient records tracking<br>(patient questionnaires and/or calls, GP records, hospital/ED records)
Death<br>during follow-up of 6 months	Patient records tracking<br>(GP records, hospital records)
If applicable: Reasons for readmission, ED visits, GP encounters or death	Patient records tracking<br>(patient questionnaires and/or calls, GP records, hospital/ED records)
Number of drugs<br>at discharge	Hospital records at discharge<br>(patient records tracking and/or calls, GP records)
Anatomical Therapeutic Chemical (ATC) classes of the drugs prescribed/de-prescribed<br>at discharge	Hospital records at discharge<br>(patient records tracking and/or calls, GP records);<br>consecutive classification at study center
Proportion of potentially inappropriate medications (PIMs)<br>at discharge	Hospital records at discharge<br>(patient records tracking and/or calls, GP records);<br>consecutive classification at study center based on 2012 Beers criteria (1) and PRISCUS list (16)
Number of drugs<br>at 1, 3 and 6 months after discharge	Patient records tracking and/or calls<br>(GP records)
ATC-codes of the drugs prescribed<br>at 1, 3 and 6 months after discharge	Patient records tracking and/or calls<br>(GP records);<br>consecutive classification at study center
Proportion of potentially inappropriate medications (PIMs)<br>at 1, 3 and 6 months after discharge	Patient records tracking and/or calls<br>(GP records);<br>consecutive classification at study center based on 2012 Beers criteria (1) and PRISCUS list (16)
Patients’ quality of life<br>at discharge	EQ-5D-3L-scale

Patients’ quality of life<br>at 1, 3 and 6 months after discharge	EQ-5D-3L-scale

The following covariates will be used for balanced cluster allocation and subgroup analyses:

**Covariates**	**Measurements**
Patient characteristics<br>(age, sex)	Patient declaration at inclusion
Hospital type<br>(A-D[1]; University or Non-University)	Questionnaire/interview with decision maker<br>at recruitment of clinic
Type and size (number of beds)<br>of hospital clinic/ward/unit	Questionnaire/interview with decision maker<br>at recruitment of clinic

5.3 Process Evaluation Outcomes

**Outcomes**	**Measurements**
HP-GP interaction/contacts with regard to the impending discharge	HP records at discharge
Frequency of GPs’ utilization<br>of communication offered from HPs	Senior HP records after inclusion of last patient
Ratings of feasibility/acceptance<br>among HPs	Questionnaires for senior HPs to be completed after inclusion of last patient, with 5-point-Likert scale in regard to acceptance and feasibility among HPs, e.g.:<br>• “The tool was well applicable in my daily work.”<br>• “The tool was useful in regard to deprescribing.”<br>• “I will use the tool also after the study.”<br>• “I would recommend the tool to my colleagues.”<br>• “The reactions of my patients to the tool were positive.”
Barriers and enablers of deprescribing among senior HPs	Semi-structured telephone calls or focus group interviews with/of senior HPs
Dropout rates<br>of HPs, GPs and patients	Study register data

[1] Clinical units of Swiss hospitals belong to one of four categories A, B, C or D, depending on their size, institution and the quality of further education provided.

6 STUDY DESIGN AND COURSE OF STUDY

6.1 General Study Design and Justification of the Design

To test our hypothesis we designed a cRCT with a "usual care" control group. The intervention is an educative training session of senior HPs responsible for patients' discharge from hospital combined with specific changes in the discharge procedure and records, as described in more detail in section 8.1.1, while the parallel control group will get usual care at discharge.

As several participating HPs might work at the same hospital ward, there is a non-negligible danger of contamination bias by information exchange among HPs. This potential contamination effect would decrease the overall effect of the intervention, thus leading to an underestimation of the effect. Therefore, we plan to randomize on the level of the senior HPs in charge of the clinical units/hospital wards (instead of the single HP or patient level) to either the intervention or the control group. In order to reduce contamination, we will allocate all senior HPs working in the same clinic/ward to the same study arm. Even so, our study design is still conservative in the sense that we will tolerate potential contamination effects resulting from senior HPs working in (different clinics/wards of) the same hospital as long as there will be no switching of HPs from the intervention arm to the control arm of the study.

In order to estimate the variation between clusters we plan a run-in phase lasting one month to assess usual medication at discharge. Procedures and materials will be pilot-tested before starting the actual trial. Data from the pilot test will be analyzed using a mixed methods approach for qualitative (e.g. awareness, perception) and quantitative results (e.g. ratings on Likert scales).

The cluster-randomized clinical trial will then take place in several northern and central Swiss hospital wards of different levels (primary to tertiary hospitals) and involve the HPs, GPs and their patients. The trial was designed as a **prospective parallel controlled double-blind single-center study** with a patient **recruitment phase of 4 months** and a **follow up of 6 months**. The flow chart in section 9.1 gives an overview of the study design.

The envisioned number of 42 clusters (i.e. senior HPs; see sample size calculation in section 11.2) will be randomly allocated to two equally sized trial arms by an administrative member of the study staff using a software random number generator. After the training of the senior HPs, patient recruitment will be carried out by the senior HPs themselves or on their behalf by internship physicians or nurses; a person responsible for recruitment will be designated for every participating clinic/ward. All patients recruited by an individual senior HP or his delegates (on average 50 patients per senior HP) will be assigned to the respective senior HP’s study arm, thus defining one cluster.

The participating physicians will neither be informed what study arm they belong to nor given specific details of the intervention of the other study arm. Similarly, the patients will not know whether their discharge follows the study (verum) or the “usual” (control) procedures.

The study and control interventions are explained in more detail in subsections 8.1.1 and 8.1.2, and data collection is laid out in sections 9.2 and 9.3. After follow up is completed for all included patients, the HPs in the control group will be debriefed and offered access to and training in the study instrument.

In a process evaluation study carried out in parallel to the RCT we will explore barriers and enablers in terms of acceptance, feasibility and future use of the study intervention, using semi-structured telephone calls or focus group interviews and questionnaires. As a secondary analysis using the original data, a cost analysis will be carried out on the basis of present medication prizes, and costs saved by spared drugs and avoided hospitalizations will be estimated.

6.2 Study Duration and Study Schedule

**Time (year)**	**2016**	**2017**			**2018**				**2019**				**2020**
Quarter	1-4	1-2	3	4	1	2	3	4	1	2	3	4	1-4
Writing proposals and ethics committee proposal													
Designing intervention													
Pilot test													
Adaptation RCT to pilot results													
Recruitment RCT													
Data collection RCT													
Data analysis RCT													
Cost analysis													
Process evaluation study													
Communication, dissemination													
Reporting, paper writing													
Steps, milestones					A					B		C	D

Milestones of the project:

- A Study design, protocol and ethics proposal, pilot test of material and procedures in hospital setting
- B Cluster-randomized controlled trial
- C Cost analysis study
- D Process evaluation study (barriers, facilitators among HPs, GPs and patients) and communication/dissemination

6.3 Methods of Minimizing Bias

Most of the outcomes are objectively measurable parameters (times and rates) whose collection does not need to rely on the patients' recollection alone but can fall back on systematic documentation at different levels (e.g. GP records). This redundancy of sources can be exploited to increase data quality/reliability, for example in cases of vague or inaccurate information from the patients themselves. The remaining outcomes will be collected either using validated scales (EQ-5D-3L) or tried-and-tested methodology (semi-structured interviews and telephone calls) from previous studies (32, 52).

In order to minimize the risk of mutual interference between clusters in the intervention and control arms, the teaching sessions for senior HPs will be held as separate events, and the participating physicians will be discouraged from discussing aspects of the study with peers who take part in the study as well.

6.3.1 Randomization

Randomization will take place on the level of the participating senior HPs (= clusters) whereby senior HPs working in the same clinic/ward/hospital unit will be jointly allocated to the same study arm. Intern physicians will be assigned deterministically to their senior HPs' study arms. The consecutive inclusion of eligible patients by their HPs will not involve further randomization either as any included patient will be allocated to the trial arm (intervention or control group) of her/his recruiting physician.

Randomization on the cluster level to equally sized treatment arms will be performed using a software random number generator by a member of staff not involved in planning of the study nor the recruitment of participants. Greatest possible balance between the study arms, in terms of the covariates listed in section 5.2, will be achieved through a customized minimization procedure described in more detail in section 7.3.

6.3.2 Blinding Procedures

The study format is double-blind in the sense that neither will the discharging HPs know which of the two interventions they provide nor will the patients know whether they receive the verum or the control intervention. Both HPs and patients will be informed that the trial aims at studying and improving the procedures at and after hospital discharge of multimorbid patients, but they will be unaware of procedural details pertaining to the study arm to which they do not belong.

The non-blinded investigators and the biometrician at the study center will not be involved in measuring outcomes.

6.4 Unblinding Procedures (Code break)

An emergency code break will be available to the investigators at the study center. This code break should be opened only in emergency situations when the identity of the investigational intervention must be known to the investigator in order to provide appropriate medical treatment. However, since the patients' clinical history files will contain all relevant medical information including, in particular, any changes of the patients' medication plans, we do not expect the necessity of unblinding to occur except in the event of adverse events (AEs) in the intervention group leading to early termination of the study. In this case, all participants would be unblinded and the HPs informed about their study group affiliation.

7 STUDY POPULATION

7.1 Eligibility Criteria

We focus on chronically ill, multimorbid patients in hospitals of all types, levels and legal structures in northern, eastern and central Switzerland.

7.1.1 Inclusion Criteria

A prescription of five or more drugs is considered a characteristic for multimorbidity. Multimorbid elderly patients incapable of judgement (e.g. patients with dementia) are a clinical reality and should therefore be included in our study for increased representativity. Moreover, like any other patient they can personally benefit from prolonged readmission times, the result hoped for from our study intervention.

Based on this rationale, patients fulfilling all of the following inclusion criteria may be enrolled in the study:

Inclusion criteria:

- In-hospital patient at the time of inclusion
- Male or female of 60 years or older with five or more drugs prescribed
- Signed informed consent or – in case of a patient incapable of judgement – fulfillment of the criteria laid down in HFG Art. 24, 1a.-c. with a legal representative according to ZGB Art. 378

7.1.2 Exclusion Criteria

Hospitals who took part in the Swiss national pilot project "progress! Sichere Medikation an Schnittstellen" will not be considered for participation in the study (but may be involved in the pilot test). On the patient level, any one of the following exclusion criteria will lead to exclusion from the study:

Exclusion criteria:

- End-stage disease with a life expectancy below three months
- Cognitive inability to follow study procedures neither independently nor with assistance

7.2 Recruitment and Screening

For the adequate number of HPs and patients to be included in the trial see the sample size calculations in section 11.2.

Different hospitals – including but not limited to the Kantonsspital Baden, Kantonsspital Glarus, Kantonsspital Graubünden Chur (medical clinic), See-Spital Horgen, Spital Lachen, Spital Limmattal Schlieren, Spital Linth Uznach, Spital Männedorf, Kantonsspital Münsterlingen, Spital Muri, Spital Schwyz, Kantonsspital St. Gallen, Stadtspital Triemli Zurich, Stadtspital Waid Zürich, Kantonsspital Winterthur (polyclinic, surgical ward), Spital Zollikerberg, UniversityHospital Zurich (clinics for gastroenterology, pneumology and geriatrics) – will be contacted on the medical management level with detailed written information about the study and an invitation to participate. Should the targeted number of senior HPs be missed due to a low response rate, remainders will be sent out. If, on the other hand, the response exceeds the actual demand then a random selection of senior HPs will be drawn and the remaining respondents will be waitlisted to compensate for potential withdrawals.

The in-hospital recruitment of patients will be performed by HPs and/or nurses; a person responsible for recruitment will be defined in every clinic or ward. The patients' life expectancies and cognitive abilities will be assessed as part of the recruitment process. Patients will be recruited during four months or until the envisaged number of participants per cluster (senior HP) is reached, and follow up will last for six months for each patient.

7.3 Assignment to Study Groups

Clusters will be assigned randomly to the two equally sized study arms as soon as the required number of clusters is reached. Given the relatively small number of clusters (see sample size calculation in section 11.2) and since we expect considerable variation in hospital types and clinic sizes, careful consideration needs to be given to balancing the covariates listed in section 5.2. For the sake of simplicity in a setting where there is no need for overly sophisticated sequential allocation algorithms and in order not to sacrifice too much randomness for balance, we chose the following randomization approach:

- In a first step, simple randomization (the "sample" command of the statistical software package "R") will be used to generate N complete and fully random allocation schemes, imposing no constraints regarding balance except for equally sized study arms.
- In the second step, a set of n (<< N) schemes with the lowest imbalances will be identified, using a similar criterion as in minimization techniques (for an overview see for e.g. (30)), or, alternatively, all schemes with a criterion value below some prespecified cut-off value.
- Finally, exactly one of these remaining schemes will be selected at random.

The whole allocation process will be carried out by an independent person not involved in planning the trial nor delivering the intervention nor collecting outcomes. The investigators will only receive the final allocation scheme from the person responsible for allocation. This list of senior HPs belonging to the intervention and control groups, respectively, will be kept at the study center.

7.4 Criteria for Withdrawal/Discontinuation of Participants

A limited number of dropouts among HPs and patients has been taken into account when assuming a censoring probability of 40% for sample size calculations (see section 11.2).

Any patient withdrawing his consent will be asked for reasons; individual follow up will be discontinued and his accumulated data will be evaluated in coded form. Patients revoking their consent will be replaced in the ongoing recruitment process while possible.

Discontinuation of participants due to safety reasons, failure of participants to adhere to protocol requirements, disease progression, or abrupt termination of the study intervention is not possible given that the intervention is one-time only and non-continuing.

8 STUDY INTERVENTION

8.1 General Information and Administration of Interventions

8.1.1 Study Intervention

The intervention takes place on different levels with different target populations:

a. *Teaching session ("Teach-the-Teachers") for the senior HPs in charge of postgraduate training and supervision of the interns (assistant physicians)*

The purpose of this training of two hours duration is to integrate the discharge procedure (see below) into the daily work of the HPs during the study period. Consecutively, the senior HPs will teach the interns how to apply the structured discharge procedure. This will guarantee the consistency of the intervention even if the fluctuation among interns is high due to organized job rotations within hospitals.

The teaching session will address the following items:

- How to identify patients eligible (possibly in collaboration with nurses)
- How to handle the in- and exclusion criteria
- How to apply the medication review tool (discharge checklist in forms 5b and 5c, see list of study instruments in appendix 18.1) which has been previously tested (15, 32) to the medication plan of the patient before discharge by the HP
- How to involve the senior HP responsible for the interns' supervision in the medication review, depending on the clinic's/wards' usual discharge procedures
- How to involve patients in the changes suggested by the HP
- How to create a discharge medication plan for the patient, after the decision is taken
- How to involve the GPs in the post-discharge phase. An offer for the GP to call the HP in order to discuss the medication plan is prepared on the discharge medication plan. This should guarantee a consensus between HP and GP in order to increase the GPs' adoption of the changes suggested on the long term
- How to deal with the different data collection forms

b. *The intervention at discharge*

The intern HP performs a critical review of the medication plan, supervised by his senior HP, discusses the results of this review and his suggestions with the patient and creates a discharge medication plan to be used by the patient. The patient is encouraged to consult his GP within seven days.

Furthermore, the intern HP communicates all medication changes (cessation of specific medication, change of dosage, introduction of new drugs) to the patient's GP in written form, accompanied with an invitation to the GP to discuss any medication changes (forms 5b and 5c, see appendix 18.1).

To ensure correct and complete implementation of the discharge procedure the intern HP documents these steps in writing, using a specifically designed checklist (also to be found in forms 5b and 5c, appendix 18.1).

At present, the above mentioned CRFs are to be understood as samples, since the optimal data collection and transmission strategy at discharge will be worked out individually with each clinic in order to minimize the additional documentation workload of the HP.

8.1.2 Control Intervention

The control group will receive “usual care”, meaning that the HPs will discharge all patients following the established discharge procedures of their respective clinics. In a two-hour presentation, a minimum of necessary information will be given to the participating HPs concerning multimorbidity as well as legal and organizational aspects of the study (eligibility of patients, informed consent, handling and disclosure of patient data).

8.2 Compliance with Intervention

Patients are encouraged to consult their GPs within seven days after discharge but failure to do so is not considered to be in violation of the study regimen, as GP consultation is not mandatory. Therefore, no specific procedures are foreseen in such cases.

8.3 Data Collection and Follow-up for Withdrawn Participants

In case of pending responses from patients at 1, 3 or 6 months after discharge the patients will be contacted by phone or in writing and invited to provide any missing data.

Any patient withdrawing his consent will be asked for reasons, and his accumulated data will be evaluated in coded form. Both the patients’ right to withdraw consent at any point of time and the consequences thereof are explained in the patient information sheet.

8.4 Concomitant Interventions

Patients may receive any drug or other therapy for any illness before and during the study, and no specific rules will prohibit any such treatment. All treatments will be documented in the patients’ personal medical records as usual.

Personal, social, professional or economic circumstances that might affect a patient’s state of health are captured globally within the QoL-questionnaires.

9 STUDY PROCEDURES

9.1 Study Flow Chart/Table of Study Procedures and Assessments

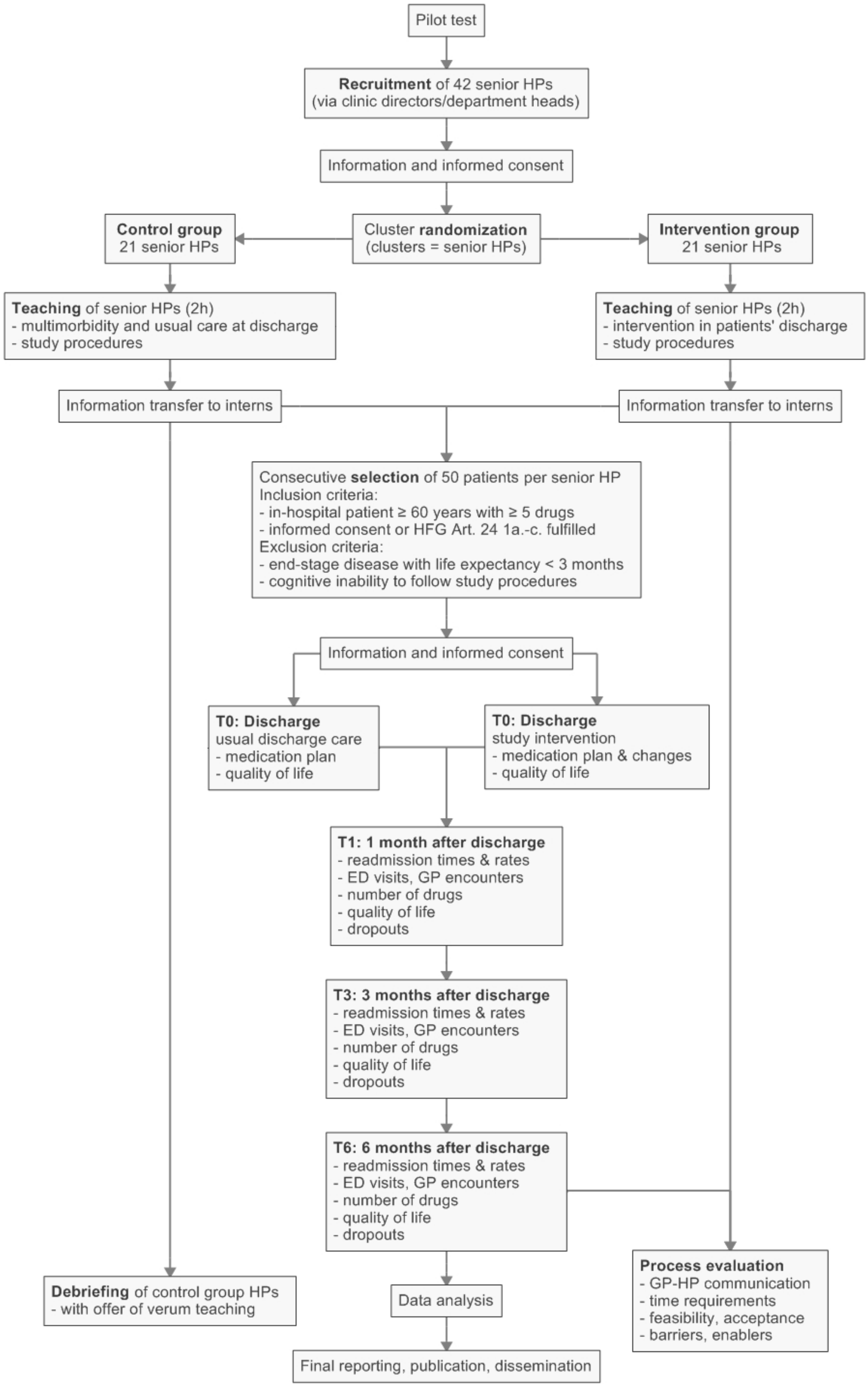

Study Period	HP Recruitment and Teaching		Hospital Stay		Post Hospital Follow Up			Eval. and Dissem.
Time Intervall	01/2018-06/2018		03/2018-03/2019					10/2018 -12/2020
Point in Time (Months after Discharge)				0	1	3	6	
**Study Stage**	**R**	**T**	**A**	**D = T0**	**T1**	**T3**	**T6**	
HP Recruitment	x							
HP Information and Informed Consent	x							
Clinc Characteristics	x							
Randomization	x							
HP Teaching		x						
Patient Selection and In-/Exclusion			x					
Patient Information and Informed Consent			x					
Biometric Patient Data			x					
Medication Plan				x				
Changes in Medication				x				
Readmission with Date (if applicable)					x	x	x	
ED Visits/GP Encounters					x	x	x	
ATC-Codes				x				
Number of Drugs				x	x	x	x	
Quality of Life				x	x	x	x	
Frequency of GP-HP Communication					x			
Time for Applying Intervention				x				
Dropout Rates					x	x	x	
Feasibility/Acceptance and Barriers/Enablers								x
Debriefing of Control HPs								x
Data Analysis								x
Final Reporting/ Publication/Dissemination								x

Study stages: R = Recruitment, T = Teaching, A = Admission, D = T0 = Discharge, T1/2/3 = 1/2/3 months after discharge

9.2 Assessments of Outcomes

The assessment of all outcomes is laid out in sections 5.1-5.3 and 9.3. Additional details and all assessment tools can be found in the appendices.

9.2.1 Assessment of Primary Outcome

Possible readmission and the respective dates (or non-readmission, respectively) will be collected from the patients in writing at 1, 3 and 6 months after discharge using paper case report forms. In case of outstanding responses the study team will contact (in the following order) patients, relatives, GPs or hospitals by phone or in writing in order to complete the missing data.

9.2.2 Assessment of Secondary Outcomes

Readmission rates will be calculated from primary outcome data, and the number of ED visits and GP encounters will be collected in the same way as the primary outcome data.

Patients will be requested to submit a copy of their current medication plans together with the completed QoL-questionnaires at discharge and after 1, 3, and 6 months to the study center, from which ATC-codes and the number of drugs will be compiled. Again, any attempt will be made to collect missing data by phone calls or in writing from patients, relatives, GPs and hospitals. This procedure will also capture deaths and dropout rates of patients.

Data regarding GP-HP contacts and communication before and after discharge and ratings of feasibility and acceptance will be collected from HP records and questionnaires. Semi-structured telephone interviews will be used to assess barriers and enablers of deprescribing among HPs and patients.

9.2.3 Assessment of Covariates

Patient characteristics (sex, age) will be collected together with the patients' informed consent.

9.2.4 Assessment of Safety Outcomes

9.2.4.1 Serious Adverse Events

For the definition of serious adverse events (SAEs) see section 10.1. Inpatient treatment and death are assessed within this study as primary or secondary outcomes, respectively, and will be recorded retrospectively.

9.2.5 Assessments in Participants who Prematurely Stop the Study

Patients who withdraw their consent or prematurely stop the study for subjective reasons will remain in the care of their attending GPs. No further safety outcomes will be recorded within the study.

9.3 Procedures at Each Study Stage

9.3.1 R: Recruitment of HPs

- Invitation of hospitals to participate in study
- Provision of written information about study for senior HPs and collection of their informed consent
- Collection of senior HP data and descriptors of their hospitals and clinical units (i.e. hospital and clinic type and size)
- Randomization of senior HPs

9.3.2 T: Teaching of HPs

- Teaching sessions for senior HPs according to trial arm

9.3.3 A: Patient Admission

- Screening of participants according to in- and exclusion criteria
- Provision of written information about study and collection of the patients' informed consent
- Collection of patient characteristics (age, sex)

9.3.4 D = T0: Patient Discharge (Baseline)

- Discharge procedure: In accordance with the checklist (see forms 5b and 5c, appendix 18.1) in intervention arm; "usual discharge care" in control arm
- Communication of
    - changes in medication (including offer of discussion) and (provisional) discharge report (including medication plan) in the intervention arm or
    - (provisional) discharge report (including medication plan) in the control arm

  to the patients' GPs
- Submission of
    - discharge checklist and changes in medication, informed consent form, QoL-questionnaire and medication plan in the intervention arm or
    - informed consent form, QoL-questionnaire and (provisional) discharge report (including medication plan) in the control arm

  to the study center

9.3.5 T1: 1 Month After Discharge

- Reporting of readmission, ED visits and GP encounters
- Submission of current medication plan and QoL-questionnaire to study center

9.3.6 T3: 3 Months After Discharge

- Reporting of readmission, ED visits and GP encounters
- Submission of current medication plan and QoL-questionnaire to study center

9.3.7 T6: 6 Months After Discharge

- Reporting of readmission, ED visits and GP encounters
- Submission of current medication plan and QoL-questionnaire to study center

10 SAFETY

10.1 Definition of Serious Adverse Events

Within this study, a serious adverse event (SAE) is defined as any untoward medical occurrence in a patient happening after the intervention which:

- requires inpatient treatment or extends a current hospital stay
- results in permanent or significant incapacity or disability
- is life-threatening or results in death

10.2 Recording and Assessment of Serious Adverse Events

Safety outcomes (readmission, ED visits, GP encounters, death) form an integral part of the study design and will be collected retrospectively at all follow up stages (1, 3 and 6 months after the intervention).

A possible causal dependence of individual SAEs on the study intervention will be assessed by the study team according the following definitions:

**Unrelated:**	• The event started in no temporal relationship to the medical intervention applied and<br>• The event can be definitely explained by underlying diseases or other situations.
**Related:**	• The event started in a plausible temporal relationship to the medical intervention applied and<br>• The event cannot be definitely explained by underlying diseases or other situations.

10.3 Reporting of Serious Adverse Events

All SAEs will be summed up in the annual safety report (ASR) and submitted to the CEC. The ASR will contain a summary of the events including severity and causal relationship to the intervention and implications on the safety of participants. The enclosed letter to be provided with the ASR will contain a short summary of the status of the clinical trial.

10.4 Follow up of (Serious) Adverse Events

Any participant terminating the study (either regularly or prematurely) with a reported ongoing SAE will remain in the medical care of his attending physician who will provide clinical monitoring until a final evaluation after termination of the SAE.

Follow-up investigations may also be necessary according to the investigator’s medical judgment even if the patient has no SAE at the end of the study.

Information related to clinical monitoring and follow-up investigations will be noted in the source documents.

10.5 Urgent Safety Measures

Any finding or result of the trial that might jeopardize all participants' safety and requires immediate preventive or corrective action will be reported to the CEC within 48 hours.

10.6 Early Termination of the Study due to Safety Concerns

The sponsor-investigator can terminate the study at any point of time in case of substancial ethical or participant safety concerns associated with continuing the study.

11 STATISTICAL METHODS

11.1 Hypothesis

- A simple medication review tool in combination with a defined communication strategy at hospital discharge leads to longer hospital readmission times compared to usual care at discharge (control group)
- and has the potential to improve the patients' health outcomes and quality of life (see secondary outcomes in section 5.2).

11.2 Determination of Sample Size

We modeled hospital readmission time by fitting an exponential survival curve on published readmission rates (48, 46) and internal data from the UniversityHospital Zurich. Extending readmission time by 25% was considered a relevant effect of the intervention. In the fitted model, this translates into an increase of the median readmission time by 23.2 days (from 92.7 days to 116.8 days) and corresponds to a hazard ratio of 0.80 between intervention and control group participants. This is equivalent to a decrease of 18.2% in readmission rates after 30 days (from 20.1% to 16.4%).

Based on this hazard ratio and assuming a two-sided $\alpha = 5\%$, a power of $1-\beta = 80\%$, an intraclass correlation coefficient (ICC) of 0.02 as in (18), 40% overall censoring probability and equally sized clusters of 50 patients, we calculated a sample size of 21 clusters and 1’050 individuals per trial arm. Thus, 42 senior HPs and 2’100 patients in total will need to be included in order to observe a relevant effect of the study intervention with sufficient power.

11.3 Planned Analyses

All statistical analyses will be carried out by the study team experienced in the conduct of cluster-randomized trials.

11.3.1 Datasets to be Analyzed, Analysis Populations

The analyses of primary and secondary outcomes will follow the intention-to-treat principle. Per-protocol analyses will be performed within the scope of sensitivity analyses. In the intervention group we will carry out exploratory analyses to identify factors related to drug deprescription.

11.3.2 Primary Analysis

Descriptive statistical methods will be used to describe the study population, including dropouts and losses to follow up. Baseline characteristics of the intervention and control groups will be calculated with corresponding 95% confidence intervals where applicable.

The primary outcome will be compared between groups using Kaplan-Meier and log rank tests. To compare factors which may affect readmission, Cox regression (proportional hazards model) will be used considering hospital clinics/units as clusters. The multivariable model will include patient characteristics as factors as well as all covariates used for balanced allocation of clusters to the study arms, and subgroup analyses may be carried out for such factors or covariates.

For secondary outcomes, parametric (*t*-test) or non-parametric tests (χ^2- and Wilcoxon test) will be used where appropriate.

11.3.3 Secondary Analyses

A mixed methods approach will be used for the pilot test to get quantitative (e.g. ratings on a 5-point Likert scale) as well as qualitative (e.g. awareness, perception) results.

In the intervention group, determinants associated with a change of medication will be identified and investigated by exploratory, multivariate regression analysis.

As a secondary analysis using the original data, a cost analysis will be carried out on the basis of present medication prizes, and costs saved by spared drugs and avoided hospitalizations will be estimated.

For process evaluation purposes we will explore barriers and enablers in terms of acceptance, feasibility and future use of our intervention, using semi-structured interviews or questionnaires within focus groups.

11.3.4 Interim Analysis

Successful recruitment might be lower than expected. Therefore, we plan a first randomization step after the inclusion of 20 clusters (10 clusters per trial arm), i.e. 50% of the full sample size, with a subsequent interim analysis of this first set of clusters, while continuing with further recruitment. This should open the option for a partial publication/dissemination of results even for the case of a poor recruitment success. If the recruitment success will be closer to the sample size yielded, this two step randomization approach and the interim analysis will be dropped.

11.3.5 Deviations from the Original Statistical Plan

Necessary deviations from the original statistical plan will be duly substantiated and reported to the CEC at the earliest possible date.

11.4 Handling of Missing Data and Dropouts

Dropouts will be recorded with reasons. A possible influence of the intervention on dropout probability will be analyzed by comparing dropout rates and dropout reasons between the two study arms. Missing data will be handled by multiple imputation, and in order to assess the imputation effect an additional complete case sensitivity analysis will be carried out.

12 ELIGIBILITY OF THE PROJECT SITES

The Institute of Primary Care of the UniversityHospital Zurich will serve as the sole study center, while hospitals of all types, levels and legal structures are eligible as project sites. No study-specific infrastructure is needed at the project sites.

13 DATA QUALITY ASSURANCE AND CONTROL

The Sponsor-Investigator warrants by implementing and maintaining quality assurance and quality control systems that the trial is conducted and all data is generated, documented (recorded), and reported in compliance with the protocol, GCP, and applicable regulatory requirements. Monitoring and Audits will be conducted during the course of the study for quality assurance purposes.

The study will strictly follow the protocol. If any changes become necessary, they will be laid down in an amendment to the protocol. All amendments of the protocol will be signed by the Sponsor-Investigator and, if essential, submitted to CEC.

13.1 DATA HANDLING AND RECORD KEEPING

All electronic records and data will be kept under password protection at the Institute of Primary Care of the UniversityHospital Zurich.

13.1.1 Case Report Forms

The senior HPs will document the participation of each patient in an enrolment log (form 5a in the list of study instruments in appendix 18.1). One file of paper case report forms (CRFs) will be kept for each enrolled study participant, to be filled in completely and accurately with all relevant data pertaining to the participant during the study.

All requested information in the CRF should be completed in a neat legible manner. Use of a black ball pen will be recommended to ensure clarity of reproduced copies in the CRF. All corrections in a paper CRF shall be made in a way that does not obscure the original entry. The correct data must be inserted, dated and initialed by the investigator. Data that are not available or not done should be made clear by adding NA or ND. A declaration ensuring accuracy of data recorded in the case report forms must be signed by the investigator.

For data and query management, monitoring, reporting and coding the clinical data management tool “OpenClinica” will be used. The investigator will ensure that all data in the course of the study will be entered completely and correctly in the database. Corrections may only be performed by the investigator or by other authorized and identifiable persons. A list of all authorized persons with initials and signatures will be filed in the study site file and the trial master file, respectively. In case of corrections the original data entries will be archived in the system and can be made visible. For all data entries and corrections date, time and person who is performing the entries will be generated automatically.

Both the paper CRFs and the database will be kept current to reflect participant status at each phase during the course of study. Participants will be identified in the CRFs and the database by appropriate identification codes.

Essential documents including any patient files and source data will be retained for at least 10 years after the regular end or a premature termination of the study (ClinO Art. 45).

13.1.2 Specification of Source Documents

All original documents relating to the study (including but not limited to CRFs, logs, medical records from and correspondence with other departments or hospitals, if participant visited any during the study period or the post study period) as well as the medical history of the participants are considered source data. Source data must be available at the sites (study center, hospitals or GPs’ offices) to document the existence of the study participants and substantiate the integrity of study data collected.

The following information (at least but not limited to) will be included in the source documents:

- Patient data (age, sex)
- Inclusion and exclusion criteria details
- Participation in study and signed and dated informed consent forms
- Medical history
- Key efficacy and safety data (as specified in the protocol)
- Related SAEs
- Results of relevant examinations
- Reason for premature discontinuation
- Randomization and identification numbers

13.1.3 Record Keeping/Archiving

All study data will be archived at the study center (Institute of Primary Care, UniversityHospital Zurich) for a minimum of 10 years after study termination or premature termination of the clinical trial.

13.2 Data Management

Relevant study data from paper log files and the patients' CRFs will be transferred to a digital database by a member of staff at the study center using the data capture software "OpenClinica". A second member of staff will supervise data entry into the database and both manual and software-assisted plausibility-checks will be applied to ensure data integrity.

The members of the study team will have exclusive access to the database stored on a central server of the UniversityHospital Zurich. "OpenClinica" will be used for data management, and periodic backups will be run according to the backup policy and schedules of the UniversityHospital Zurich.

13.3 Routine Monitoring

Monitoring the project sites prior to the start and during the course of the study will help to follow up the progress of the clinical study, to assure utmost accuracy of the data and to detect possible errors at an early stage. Monitoring will be done primarily by phone and if needed by visits at the study/project sites.

All original data including the patient files (in particular all written informed consents and the CRFs), progress notes and copies of laboratory and medical test results must be available for monitoring. The accuracy of the data will be verified by reviewing the above referenced documents. The study center will collaborate with the Clinical Trials Center (CTC) of the University Hospital Zurich to ensure monitoring.

13.4 Audits and Inspections

A quality assurance audit/inspection of this study may be conducted by the CEC. The quality assurance auditor/inspector will have access to all medical records, the investigators' study related files and correspondence, and the informed consent documentation that is relevant to this clinical study.

The investigators will allow the persons being responsible for auditing or the inspection to have access to the source data/documents and to answer any questions arising. All involved parties will keep the patient data strictly confidential.

13.5 Confidentiality, Data Protection

Direct access to source documents will be permitted to the CEC for purposes of monitoring, audits and inspections, during and after conclusion of the study.

For contact maintenance and case tracking (e.g. in case of adverse events), the patients' identities will be known to a study nurse not involved in the analysis of the patient data. The patient names will not be accessible to the scientific study staff.

All electronic data, including interview transcripts, will be stored under password protection on secure network drives of the UniversityHospital Zurich.

14 PUBLICATION AND DISSEMINATION POLICY

The study protocol will be submitted for publication in the BioMed Central journal “Implementation Science” (alternatively: “Trials”) before completion of patient recruitment.

After the statistical analysis of this trial the sponsor will make every endeavor to publish the data at medical conferences and in (a) medical journal(s). The results of the study will also be communicated to interested study participants, hereto using comprehensible language for patients and an appropriate scientific format for hospital and primary care physicians.

15 FUNDING AND SUPPORT

The following table gives estimates of the costs in CHF for individual stages/years of the study:

Year	2017	2018	2019/2020
Writing proposals, pilot test of material and procedures<br>• Scientific collaborator, 25%, 1 year<br>• Study nurse, 50%, 6 months	50’000<br>27’000<br>22’500		
Cluster-randomized clinical trial<br>• Scientific collaborator, 50%, 2 years<br>• Dissertant, 50%, 1.5 years<br>• Study nurse, 50%, 2 years<br>• Equipment, logistics, technical support, fees		135’000<br>55’000<br>25’000<br>45’000<br>10’000	112’500<br>55’000<br>12’500<br>45’000
Implementation evaluation and cost analysis<br>• Scientific collaborator, 50%, 6 months, and 100%, 3 months<br>• Dissertant, 50%, 1 year<br>• Study nurse, 50%, 6 months			102’500<br>55’000<br>25’000<br>22’500
Communication and dissemination			10’000
Total per year	**50’000**	**135’000**	**225’000**

The overall costs of the study are therefore estimated to amount to **410’000 Swiss francs**.

Funding of the project is provided within the National Research Programme "Smarter Health Care" (NRP 74) by the Swiss National Science Foundation with an amount of CHF 393'268.00 (request number 407440_167193 / 1, granted on Dezember 7, 2016), payable in four yearly tranches:

	2017	2018	2019	2020	Total
Research funds	5’000	5’000	5’000	5’000	20’000
Salaries	103'562	111'907	64'867	44'242	324'578
Social security taxes	15'535	16'787	9'731	6'637	48'690
Total per year/grand total	**124'097**	**133'694**	**79'598**	**55'879**	**393'268**

No other support is available from sources outside the Institute of Primary Care of the UniversityHospital Zurich.

16 INSURANCE

Insurance is covered by “Versicherung für klinische Versuche und nichtklinische Versuche“ by Zürich Versicherungs-Gesellschaft AG (Policy no.: 14.970.888).

Any damage developed in relation to study participation is covered by this insurance. So as not to forfeit their insurance cover, the participants themselves must strictly follow the instructions of the study personnel. Participants must not be involved in any other medical treatment without permission of the principal investigator (emergency excluded). Medical emergency treatment must be reported immediately to the investigator. The investigator must also be informed instantly in the event of health problems or other damages during or after the course of study treatment.

The investigator will allow delegates of the insurance company to have access to the source data/documents as necessary to clarify a case of damage related to study participation. All involved parties will keep the patient data strictly confidential.

A copy of the insurance certificate will be kept in the Investigator’s Site File.

17 REFERENCES

1. The American Geriatrics Society 2012 Beers Criteria Update Expert Panel. American Geriatrics Society Updated Beers Criteria for Potentially Inappropriate Medication Use in Older Adults. J Am Geriatr Soc. 2012;60(4):616-31.

2. Anthierens S, Tansens A, Petrovic M, Christiaens T. Qualitative insights into general practitioners views on polypharmacy. BMC Fam Pract. 2010;11:65.

3. Blix HS, Viktil KK, Reikvam A, Moger TA, Hjemaas BJ, Pretsch P, et al. The majority of hospitalised patients have drug-related problems: results from a prospective study in general hospitals. Eur J Clin Pharmacol. 2004;60(9):651-8.

4. Briggs S, Pearce R, Dilworth S, Higgins I, Hullick C, Attia J. Clinical pharmacist review: a randomised controlled trial. Emerg Med Australas. 2015;27(5):419-26.

5. Budnitz DS, Lovegrove MC, Shehab N, Richards CL. Emergency hospitalizations for adverse drug events in older Americans. N Engl J Med. 2011;365(21):2002-12.

6. Clyne B, Smith SM, Hughes CM, Boland F, Bradley MC, Cooper JA, et al. Effectiveness of a Multifaceted Intervention for Potentially Inappropriate Prescribing in Older Patients in Primary Care: A Cluster-Randomized Controlled Trial (OPTI-SCRIPT Study). Ann Fam Med. 2015;13(6):545-53.

7. Costantino ME, Frey B, Hall B, Painter P. The influence of a postdischarge intervention on reducing hospital readmissions in a Medicare population. Popul Health Manag. 2013;16(5):310-6.

8. Drenth-van Maanen AC, van Marum RJ, Knol W, van der Linden CM, Jansen PA. Prescribing optimization method for improving prescribing in elderly patients receiving polypharmacy: results of application to case histories by general practitioners. Drugs Aging. 2009;26(8):687-701.

9. Fick DM, Cooper JW, Wade WE, Waller JL, Maclean JR, Beers MH. Updating the Beers criteria for potentially inappropriate medication use in older adults: results of a US consensus panel of experts. Arch Intern Med. 2003;163(22):2716-24.

10. Frazier SC. Health outcomes and polypharmacy in elderly individuals: an integrated literature review. J Gerontol Nurs. 2005;31(9):4-11.

11. Garfinkel D, Mangin D. Feasibility study of a systematic approach for discontinuation of multiple medications in older adults: addressing polypharmacy. Arch Intern Med. 2010;170(18):1648-54.

12. Gerhardt G, Yemane A, Hickman P, Oelschlaeger A, Rollins E, Brennan N. Medicare readmission rates showed meaningful decline in 2012. Medicare Medicaid Res Rev. 2013;3(2).

13. Gonçalves-Bradley DC, Lannin NA, Clemson LM, Cameron ID, Shepperd S. Discharge planning from hospital. Cochrane Database Syst Rev. 2016(1):CD000313.

14. Hanlon JT, Weinberger M, Samsa GP, Schmader KE, Uttech KM, Lewis IK, et al. A randomized, controlled trial of a clinical pharmacist intervention to improve inappropriate prescribing in elderly outpatients with polypharmacy. Am J Med. 1996;100(4):428-37.

15. Hasler S, Senn O, Rosemann T, Neuner-Jehle S. Effect of a patient-centered drug review on polypharmacy in primary care patients: study protocol for a cluster-randomized controlled trial. Trials. 2015;16:380.

16. Holt S, Schmiedl S, Thürmann PA. Potentially Inappropriate Medications in the Elderly: The PRISCUS List. Dtsch Arztebl Int. 2010;107(31-32):543-51.

17. Horwitz LI, Moriarty JP, Chen C, Fogerty RL, Brewster UC, Kanade S, et al. Quality of discharge practices and patient understanding at an academic medical center. JAMA Intern Med. 2013;173(18):1715-22.

18. Jäger C, Freund T, Steinhäuser J, Joos S, Wensing M, Szecsenyi J. A tailored implementation intervention to implement recommendations addressing polypharmacy in multimorbid patients: study protocol of a cluster randomized controlled trial. Trials. 2013;14:420.

19. Jencks SF, Williams MV, Coleman EA. Rehospitalizations among patients in the Medicare fee-for-service program. N Engl J Med. 2009;360(14):1418-28.

20. Kergoat MJ, Latour J, Julien I, Plante MA, Lebel P, Mainville D, et al. A discharge summary adapted to the frail elderly to ensure transfer of relevant information from the hospital to community settings: a model. BMC Geriatr. 2010;10:69.

21. Kripalani S, LeFevre F, Phillips CO, Williams MV, Basaviah P, Baker DW. Deficits in communication and information transfer between hospital-based and primary care physicians: implications for patient safety and continuity of care. JAMA. 2007;297(8):831-41.

22. Lau DT, Kasper JD, Potter DE, Lyles A, Bennett RG. Hospitalization and death associated with potentially inappropriate medication prescriptions among elderly nursing home residents. Arch Intern Med. 2005;165(1):68-74.

23. Lee RG, Garvin T. Moving from information transfer to information exchange in health and health care. Soc Sci Med. 2003;56(3):449-64.

24. Leitliniengruppe Hessen. Hausärztliche Leitlinie Multimedikation. Version 1.09. Deutsche Gesellschaft für Allgemeinmedizin und Familienmedizin DEGAM. 2013.

25. Jahn-Eimermacher A, Ingel K, Schneider A. Sample size in cluster-randomized trials with time to event as the primary endpoint. Stat Med. 2013;32(5):739-51.

26. Kul S, Vanhaecht K, Panella M. Intraclass correlation coefficients for cluster randomized trials in care pathways and usual care: hospital treatment for heart failure. BMC Health Serv Res. 2014;14:84.

27. Leiss W, Méan M, Limacher A, Righini M, Jaeger K, Beer HJ, et al. Polypharmacy is associated with an increased risk of bleeding in elderly patients with venous thromboembolism. J Gen Intern Med. 2015;30(1):17-24.

28. Leppin AL, Gionfriddo MR, Kessler M, Brito JP, Mair FS, Gallacher K, et al. Preventing 30-day hospital readmissions: a systematic review and meta-analysis of randomized trials. JAMA Intern Med. 2014;174(7):1095-107.

29. Levinson W, Kallewaard M, Bhatia RS, Wolfson D, Shortt S, Kerr EA, et al. 'Choosing Wisely': a growing international campaign. BMJ Qual Saf. 2015;24(2):167-74.

30. Lin Y, Zhu M, Su Z. The pursuit of balance: An overview of covariate-adaptive randomization techniques in clinical trials. Contemp Clin Trials. 2015;45(Pt A):21-5.

31. Mayring P. Qualitative Content Analysis. Forum Qualitative Sozialforschung/Forum: Qualitative Social Research. 2000;1(2):Art. 20.

32. Neuner-Jehle S, Krones T, Senn O. Systematisches Weglassen verschriebener Medikamente ist bei polymorbiden Hausarztpatienten akzeptiert und machbar. Praxis. 2014;103(6):317-22.

33. Nobili A, Licata G, Salerno F, Pasina L, Tettamanti M, Franchi C, et al. Polypharmacy, length of hospital stay, and in-hospital mortality among elderly patients in internal medicine wards. The REPOSI study. Eur J Clin Pharmacol. 2011;67(5):507-19.

34. The OPERAM project: Optimising Therapy to prevent avoidable hospital admissions in the multimorbid elderly. Available from: http://operam-2020.eu.

35. O'Mahony D, Gallagher P, Ryan C, Byrne S, Hamilton H, Barry P, et al. STOPP & START criteria: A new approach to detecting potentially inappropriate prescribing in old age. European Geriatric Medicine. 2010;1(1):45-51.

36. Perera PN, Guy MC, Sweaney AM, Boesen KP. Evaluation of prescriber responses to pharmacist recommendations communicated by fax in a medication therapy management program (MTMP). J Manag Care Pharm. 2011;17(5):345-54.

37. Reeve E, To J, Hendrix I, Shakib S, Roberts MS, Wiese MD. Patient barriers to and enablers of deprescribing: a systematic review. Drugs Aging. 2013;30(10):793-807.

38. Reich O, Rosemann T, Rapold R, Blozik E, Senn O. Potentially inappropriate medication use in older patients in Swiss managed care plans: prevalence, determinants and association with hospitalization. PLoS One. 2014;9(8):e105425.

39. Rizza A, Kaplan V, Senn O, Rosemann T, Bhend H, Tandjung R, et al. Age- and gender-related prevalence of multimorbidity in primary care: the Swiss FIRE project. BMC Fam Pract. 2012;13:113.

40. Rohrer JE, Garrison G, Oberhelman SA, Meunier MR. Epidemiology of polypharmacy among family medicine patients at hospital discharge. J Prim Care Community Health. 2013;4(2):101-5.

41. Roughead EE, Kalisch LM, Ramsay EN, Ryan P, Gilbert AL. Continuity of care: when do patients visit community healthcare providers after leaving hospital? Intern Med J. 2011;41(9):662-7.

42. Schuling J, Gebben H, Veehof LJ, Haaijer-Ruskamp FM. Deprescribing medication in very elderly patients with multimorbidity: the view of Dutch GPs. A qualitative study. BMC Fam Pract. 2012;13:56.

43. Scott IA, Hilmer SN, Reeve E, Potter K, Le Couteur D, Rigby D, et al. Reducing inappropriate polypharmacy: the process of deprescribing. JAMA Intern Med. 2015;175(5):827-34.

44. Scottish Government Model of Care Polypharmacy Working Group. Polypharmacy Guidance (2nd Edition). Scottish Government; March 2015.

45. Selby K, Gaspoz JM, Rodondi N, Neuner-Jehle S, Perrier A, Zeller A, et al. Creating a list of low-value health care activities in Swiss primary care. JAMA Intern Med. 2015;175(4):640-2.

46. Stranges PM, Marshall VD, Walker PC, Hall KE, Griffith DK, Remington T. A multidisciplinary intervention for reducing readmissions among older adults in a patient-centered medical home. Am J Manag Care. 2015;21(2):106-13.

47. Swart E. Was sagen uns Wiedereinweisungen über die Qualität der stationären Versorgung? Gesundheitswesen. 2005;67:101-6.

48. Takahashi PY, Naessens JM, Peterson SM, Rahman PA, Shah ND, Finnie DM, et al. Short-term and long-term effectiveness of a post-hospital care transitions program in an older, medically complex population. Healthc (Amst). 2016;4(1):30-5.

49. Via-Sosa MA, Lopes N, March M. Effectiveness of a drug dosing service provided by community pharmacists in polymedicated elderly patients with renal impairment--a comparative study. BMC Fam Pract. 2013;14:96.

50. Viktil KK, Blix HS, Eek AK, Davies MN, Moger TA, Reikvam A. How are drug regimen changes during hospitalisation handled after discharge: a cohort study. BMJ Open. 2012;2(6).

51. Viktil KK, Blix HS, Moger TA, Reikvam A. Polypharmacy as commonly defined is an indicator of limited value in the assessment of drug-related problems. Br J Clin Pharmacol. 2007;63(2):187-95.

52. Zechmann S, Trueb C, Valeri F, Streit S, Senn O, Neuner-Jehle S. Attitudes, beliefs and concerns towards deprescribing: a qualitative study among older, multimorbid patients with polypharmacy in Switzerland. Age and Ageing (submitted).

53. Ziaeian B, Araujo KL, Van Ness PH, Horwitz LI. Medication reconciliation accuracy and patient understanding of intended medication changes on hospital discharge. J Gen Intern Med. 2012;27(11):1513-20.

18 APPENDICES

18.1 List of Study Instruments

3a: Information letter for senior HPs

3b: Informed consent form for senior HPs
incl. hospital and clinic descriptors (type and size in terms of number of beds)

3c: Information letter for intern HPs

3d: Information letter for patients

3e: Informed consent form for patients
incl. patient characteristics (age, sex)

5a: CRF at discharge («study tool»)
version 1 with medication changes, discharge checklist and communication offer for GP

5b: CRF at discharge («study tool»)
version 2 with discharge checklist and communication offer for GP

5c: Quality of life questionnaire (at discharge)

5d: CRF at 1, 3 and 6 months after discharge (identical)
incl. readmissions, ED visits and GP encounters with reasons,
and incl. quality of life questionnaire

5e: Record form for frequency of GP-HP communication
and feasibility & acceptance questionnarie for HPs (5 point likert scale)

5f: Interview guide to assess barriers & enablers

11a: Checklist with in- and exclusion criteria for recruiters

11b: Participant list (list of included patients)
